# Evidence for contemporary and historical gene flow between guppy populations in different watersheds, with a test for associations with adaptive traits

**DOI:** 10.1002/ece3.5033

**Published:** 2019-03-29

**Authors:** Léa Blondel, Lyndsey Baillie, Jessica Quinton, Jahson B. Alemu, Ian Paterson, Andrew P. Hendry, Paul Bentzen

**Affiliations:** ^1^ Redpath Museum and Department of Biology McGill University Montreal Québec Canada; ^2^ University of British Columbia Vancouver British Columbia Canada; ^3^ Department of Biology Dalhousie University Halifax Nova Scotia Canada; ^4^ Department of Life Sciences The University of the West Indies St. Augustine Trinidad and Tobago

**Keywords:** adaptive traits, contemporary dispersal, historical gene flow, *Poecilia reticulata*, population structure

## Abstract

In dendritic river systems, gene flow is expected to occur primarily within watersheds. Yet, rare cross‐watershed transfers can also occur, whether mediated by (often historical) geological events or (often contemporary) human activities. We explored these events and their potential evolutionary consequences by analyzing patterns of neutral genetic variation (microsatellites) and adaptive phenotypic variation (male color) in wild guppies (*Poecilia reticulata*) distributed across two watersheds in northern Trinidad. We found the expected signatures of within‐watershed gene flow; yet we also inferred at least two instances of cross‐watershed gene flow—one in the upstream reaches and one further downstream. The upstream cross‐watershed event appears to be very recent (41 ± 13 years), suggesting dispersal via recent flooding or undocumented human‐mediated transport. The downstream cross‐watershed event appears to be considerably older (577 ± 265 years), suggesting a role for rare geological or climatological events. Alongside these strong signatures of both contemporary and historical gene flow, we found little evidence of impacts on presumably adaptive phenotypic differentiation, except perhaps in the one instance of very recent cross‐watershed gene flow. Selection in this system seems to overpower gene flow—at least on the spatiotemporal scales investigated here.

## 1 INTRODUCTION

Historical and contemporary patterns of dispersal and gene flow are key components shaping population genetic structure (Bohonak, 1999; Slatkin, 1987). From an historical perspective, different colonization routes and times, and different patterns of starting genetic variation can leave signatures that persist for millennia (Avise, 2000). From a contemporary perspective, recent fluctuation in population sizes and patterns of dispersal can strongly shape genetic similarity among populations (Bohonak, 1999; Endler, 1977; Slatkin, 1987). These historical and contemporary effects can interact to shape the population structure over small and large spatiotemporal scales. For example, studies of isolation‐by‐distance often find that genetic differences between populations are correlated with geographic distances, because gene flow is reduced over longer distances (e.g., Castric, Bonney, & Bernatchez, 2001; Pogson, Taggart, Mesa, & a & Boutilier, R.G.R.G.G., 2001; Crookes & Shaw, 2016). Yet, such studies also often detect discontinuities between geographically proximate populations that have different historical origins, such as different colonization events (e.g., Cuenca, Escalante, & Pinero, 2003). Our goal will be to disentangle the roles of ongoing contemporary and historical gene flow in a classic evolutionary model system—Trinidadian guppies *Poecilia reticulata* (Peters, 1859).

Gene flow, whether contemporary or historical, can play an important role in the ability of populations in different places to adapt to their local environments (Garant, Forde, & Hendry, 2007; Lenormand, 2002; Slatkin, 1987). In particular, studies focusing on *contemporary* gene flow have shown that populations exchanging more genes are often less able to diverge in adaptive traits (reviews: Räsänen & Hendry, 2008; Hendry, 2016), although other studies have found limited—or even positive—effects of gene flow on adaptive divergence (e.g., Hemmer‐Hansen, Nielsen, Frydenberg, & Loeschcke, 2007; Fitzpatrick, Gerberich, Kronenberger, Angeloni, & Funk, 2015). The importance of *historical* gene flow for ongoing adaptation is less well understood. On the one hand, we might expect the power of selection to quickly overcome any historical legacy, such that adaptive trait divergence bears little relationship to neutral genetic marker divergence (Merilä & Crnokrak, 2001). On the other hand, some studies have suggested that populations coming from different colonization events can maintain important differences in adaptive traits despite long‐term occupancy of similar environments, an effect referred to as historical contingency (Losos, Jackman, Larson, Queiroz, & Rodriguez‐Schettino, 1998; Taylor & Donald McPhail, 2000; Travisano, Mongold, Bennett, & Lenski, 1995). Although numerous studies have investigated whether phenotypic variation correlates with contemporary selection or with historical contingency (Alexander, Taylor, Wu, & Breden, 2006; Hoekstra, Krenz, & Nachman, 2005; Thorpe, Malhotra, Black, Daltry, & Wuster, 1995), the focus has not been on effects of contemporary versus historical gene flow on adaptive traits. To gain insight into such effects, we combine genetic inferences about historical and contemporary gene flow in guppies from two adjacent watersheds in Trinidad, with information on an important class of adaptive traits—male color.

Several previous studies have examined population structure in Trinidadian guppies (Alexander et al., 2006; Barson, Cable, & Oosterhout, 2009; Carvalho, Shaw, Magurran, & Seghers, 1991; Crispo, Bentzen, Reznick, Kinnison, & Hendry, 2006; Fajen & Breden, 1992; Suk & Neff, 2009; Willing et al., 2010), typically revealing that patterns of neutral genetic variation within watersheds are strongly influenced by the distance between sites and by physical barriers to movement, such as waterfalls (Crispo et al., 2006; Gomez‐Uchida, Knight, & Ruzzante, 2009; Primmer et al., 2006). In keeping with the expectation that the greatest barrier to dispersal in such systems is dry land, greater genetic differences are usually found among rather than within watersheds (Barson et al., 2009; Carvalho et al., 1991; Suk & Neff, 2009; Willing et al., 2010). However, exceptions are known wherein guppies occupying some tributaries in one watershed can show surprising genetic similarity to particular populations in other watersheds (Willing et al., 2010). These cross‐watershed affinities could reflect historical or contemporary gene flow owing to natural events, such as earthquakes or severe flooding, or human‐mediated transport. The best predictors of such cross‐watershed gene flow events are expected to be similar elevations and geographic proximity, except in the case of some longer‐distance human‐mediated transfers.

### 1.1 Our focal study system

**Figure 1:**
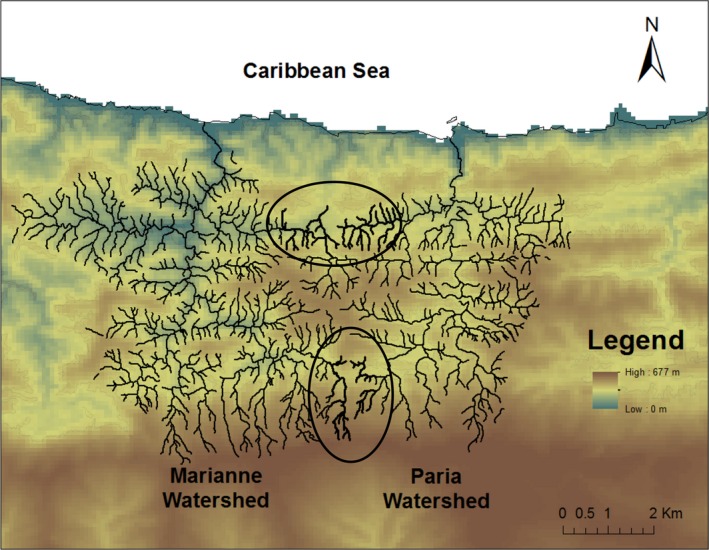
Topographic map of the Marianne and Paria watersheds. Bold and circled sections of the rivers indicate potential gene flow zones between watersheds, located at different elevations

Our work focused on guppies located on the north slope of the Northern Mountain Range in Trinidad, where multiple streams run roughly parallel to each other from the mountains over a series of waterfalls to the ocean. We studied two neighboring watersheds, the Marianne and the Paria (Figure 1). Gene flow within these watersheds is expected to be relatively high, at least in the downstream direction, as their total lengths are only 10.69 km for the Marianne and 9.22 km for the Paria. However, gene flow can be reduced in the upstream direction owing to the direction of water flow and to physical features such as waterfalls (Crispo et al., 2006). Waterfalls are present throughout the entire course of the Marianne, but there is no major waterfall along the Paria watershed that could prevent upstream migration. Gene flow would seem less likely between the two watersheds, and yet still might be possible owing to their close proximity at two elevation ranges: 50–100 m (downstream area) and 550–600 m (upstream area) (Figure 1). To examine patterns of gene flow with these expectations and possibilities in mind, we analyzed guppies from several sites for variation at 10 and 42 microsatellite loci.

To see how gene flow might relate to adaptation, we focused on male guppy color, which has a known genetic basis (Gordon, López‐Sepulcre, Rumbo, & Reznick, 2017; Lindholm & Breden, 2002) and evolves in response to sexual selection favoring conspicuousness and natural selection favoring crypsis (Endler, 1980; Reznick, Bryga, & Endler, 1990; Reznick & Endler, 1982). Most obviously, populations in different predation environments show dramatic color differences that reflect adaptation to the local balance between natural and sexual selection (Endler, 1980; Kemp, Reznick, & Grether, 2008; Millar, Reznick, Kinnison, & Hendry, 2006). These color patterns often (but not always) evolve quickly (Gordon et al., 2017; Karim, Gordon, Schwartz, & Hendry, 2007; Kemp et al., 2008), and differences among populations are stable over time (Gotanda & Hendry, 2014). Large variation also exists among populations within a given predation regime, reflecting the specific types and densities of local predators (Endler, 1978; Kemp et al., 2008; Millar & Hendry, 2012; Millar et al., 2006; Weese, Gordon, Hendry, & Kinnison, 2010), canopy cover (Grether, Millie, Bryant, Reznick, & Mayea, 2001; Schwartz & Hendry, 2010), and sexual selection (Houde & Endler, 1990; Schwartz & Hendry, 2007). Divergence in male color also has been argued to be influenced—both positively (increased divergence) and negatively (decreased divergence)—by gene flow (Endler, 1978; Fitzpatrick et al., 2015, 2017). We therefore compare patterns of gene flow with patterns of neutral genetic differentiation to infer the potential role of historical and contemporary gene flow in shaping adaptive trait divergence.

In summary, our goals were to (a) investigate population genetic structure of guppies in the two watersheds, (b) infer the existence and timing of gene flow events between sites within and between watersheds, and (c) test for associations between gene flow and differences in male color. Our interpretations proceed as follows:

1. If current watershed structure is the primary determinant of gene flow, samples should cluster by watershed; and gene flow estimates should be higher within than between watersheds. Deviations from this expectation (e.g., some clustering and inferred gene flow between watersheds) would indicate cross‐watershed genetic exchange.
2. If inferred gene flow between watersheds was due to historical—and presumably rare—events, such as earthquakes or floods, estimated divergence times between sites should be older than a few centuries. Deviations from this expectation (e.g., more recent divergence) would suggest the importance of contemporary factors, such as recent natural or human‐mediated gene flow.
3. If gene flow among sites is influencing adaptation, we expect patterns of male color divergence among sites to be associated with patterns of neutral genetic divergence. Deviations from this expectation (e.g., limited or no correspondence between neutral and adaptive divergence) would inform the extent to which local selection overcomes historical and contemporary gene flow, or would indicate genetic drift.

## 2 METHODS

### 2.1 Fish sampling

We sampled fish along the Marianne and Paria watersheds in northern Trinidad over several years (2002–2014; average of 38 individuals per year and site, min = 18, max = 50; details in Supporting Information). At each site, we used butterfly nets to capture guppies that were then transported to our laboratory in Trinidad. The fish were euthanized with a solution of tricaine methanesulfonate (MS222) and preserved in 95% ethanol for genotypic analysis. A subset of the fish was also photographed following a standard method (details below).

### 2.2 Genotypic data

Two datasets were generated using different methods implemented at different times. One dataset has fewer loci (10) but more sites (20), whereas the other dataset has more loci (42) but fewer sites (12). The term “site” refers to a specific discrete sampling location, and site numbers correspond to those reported in previous work on these watersheds (Crispo et al., 2006; Gotanda et al., 2013; Gotanda & Hendry, 2014; Millar et al., 2006; Schwartz & Hendry, 2010). We here analyze both datasets because they represent two independent efforts, with different strengths and weaknesses, to quantify genetic population structure for the same watersheds. We analyze the two datasets separately because few of the loci and only some of the sites were in common.

For the first dataset (“10loci‐20sites”), DNA was extracted using a modified glassmilk protocol (Elphinstone, Hinten, Anderson, & Nock, 2003) from fins of sampled fish. DNA was amplified by PCR and then visualized by capillary gel electrophoresis. Microsatellite markers comprised four tetranucleotide loci (*Pre9*,* Pre13*,* Pre15*, and *Pre26*: Paterson, Crispo, Kinnison, & Hendry, a. P. & Bentzen, P., 2005) and six dinucleotide loci (*Pret27, Pret28, Pret38, Pret46, Pret80*, and *G145*: Watanabe, Yoshida, Nakajima, & Taniguchi, 2003; Shen, Yang, & Liao, 2007).

For the second dataset (“42loci‐12sites”), DNA was extracted using the same method for 42 di‐ and trinucleotide microsatellite loci selected from the guppy genome (NCBI BioProject PRJNA238429). The 42 loci were multiplexed in a single PCR, and indexing sequences were subsequently added to the PCR products using a second PCR. The index PCR used oligonucleotides composed of Illumina annealing adapter sequences, a 6b index (barcode), and the Illumina sequencing primers. DNA was then sequenced on an Illumina MiSeq. Individual genotypes were characterized using megasat, a program that reads sequence files and automatically scores microsatellite genotypes. Full laboratory and bioinformatic methods are presented in Zhan et al. (2017).

micro‐checker (van Oosterhout, Hutchinson, Wills, & Shipley, 2004) was used to test for potential genotyping errors. genepop (version 4.2; Raymond & Rousset, 1995) was used to test for deviations from Hardy–Weinberg equilibrium (HWE) for each locus in each “sample” (i.e., a microsatellite dataset at a particular site in a particular year) and to test for linkage disequilibrium between loci within each sample. lositan was used to check whether loci were potentially under selection based on an *F*_ST_ outlier test (Antao, Lopes, Lopes, Beja‐Pereira, & Luikart, 2008).

We used the R software with RStudio (R Core Team, 2018; RStudio Team, 2016) to calculate basic population genetic measures. The package *pegas* (Paradis, 2010) and package* hierfstat* (Goudet, 2005) were used to calculate the number of alleles per site, as well as observed heterozygosity and gene diversities (*H*_o_ and *H*_s_, respectively). The package *hierfstat* (Goudet, 2005) was also used to calculate pairwise *F*_ST_ between samples.

structure (version 2.3.4; Pritchard, Stephens, & Donnelly, 2000) was used to infer genetic population structure and to find the appropriate number of clusters (*K)* that best explain the genotypic distribution. Three iterations were run for each *K*, from 1 to 28 or from 1 to 19 (total number of samples from the two datasets). Burn‐in length and run length of the program were each set at 100,000 using the admixture model and the correlated alleles model. We used the Evanno, Regnaut, and Goudet (2005) method implemented in structure harvester to find the best *K*. We generated structure plots using the R package *pophelper* (Francis, 2017). These analyses included multiple years of sampling for a given site so as to help assess the temporal consistency of among‐site patterns.

We estimated long‐term gene flow by calculating migration rates (*M* = *m*/*µ*) between sites using migrate (version 3.6; Beerli, 2009). In cases where a dataset included multiple years from a single site, we kept—for ease of estimation—only one year per site by choosing samples with the minimum length of time between them (i.e., temporal outliers were more likely to be excluded). We used an MCMC with Bayesian inference coalescent approach that employed a Brownian model approximating a single‐step mutation model and default values from the software. A mutation rate of 5 × 10^−4^ was chosen because it is the mutation rate commonly used in other microsatellite fish studies (Barson et al., 2009; Lippé, Dumont, & Bernatchez, 2006). For each dataset, a first run determined *F*_ST_ parameters that were then used as start values for three more runs. The number of runs was dictated by when the mean across runs was stable.

We explored recent (over the last few generations) migration rates using bayesass (version 3; Wilson & Rannala, 2003). For each dataset, we only kept one year per site, and we first adjusted the mixing parameters to meet acceptance rates. The burn‐in period of the model was then set at 1 × 10^6^, while MCMC iterations were set at 1 × 10^7^. We ran several instances of the model with different starting seeds: Results were similar among runs and so we here report only values from the first run. Model convergence was also tested using tracer (version 1.6; Rambaut & Drummond, 2013). Values calculated with this method represent the fraction of individuals in a population that are migrants derived from another population.

We used DIYABC (version 2.1.0; Cornuet et al., 2014) to estimate divergence time between pairs of sites across watersheds—the level at which such inferences were desired. This analysis was done using the 42loci‐12sites dataset, with only one year per site. For each pair of sites, we tested a simple model of two populations having diverged *t* generations in the past from a common ancestral population (Supporting Information), a reasonable approximation of a discrete cross‐watershed gene flow event. Our models thus simplify a complex scenario of watershed colonization with multiple sites but allow the comparison of pairs of sites across watersheds. The mutation model was left as the default in the program (mean mutation rate: 5 × 10^−4^). We generated 1 × 10^6^ simulated datasets to estimate the divergence time between each pair of sites. As guppies can have 2–3 generations per year (Magurran, 2005), we assumed a value of 2.5 generations per year.

### 2.3 Phenotypic data

Differences in color among guppy populations in the studied watersheds are remarkably consistent through time (Gotanda & Hendry, 2014), and so we were able to use phenotypic data (male color) from years other than the genetic data. Specifically, the data re‐analyzed here were previously published in Millar et al. (2006), wherein details are provided. In brief, we extracted color information from standardized digital pictures of male guppies. Scion Image (Scion, 2001) was used to measure body size (area, length, and depth) and each color spot (area) on the left side of the body. Each color spot was classified into a color category: orange (includes red), black, fuzzy black, yellow, blue (includes purple), green, violet‐blue, bronze‐green, and silver. For simplicity, these categories were then further grouped into three more inclusive categories: melanic (black and fuzzy black), carotenoid (orange and yellow), and structural (blue, green, violet‐blue, bronze‐green, and silver). These categories and labels are only general as, for example, the “carotenoid” colors include additional compounds influencing color (Grether, Hudon, & Millie, 1999). For the present analysis, we used—for each individual fish—the total number of spots and the relative total spot area (total spot area divided by the total body area of the fish) for each color category.

We used a MANOVA to detect differences in male color between predation regimes in the Marianne watershed. We calculated pairwise *P*_ST_ as a measure of the phenotypic (color) distance between guppies at each pair of sites. Following Phillimore et al. (2008), we used the formula $P_{\text{ST}}=\delta_{\text{GB}}^{2}/\left(\delta_{\text{GB}}^{2}+2\delta_{\text{GW}}^{2}h^{2}\right)$, where $\delta_{\text{GB}}^{2}$ and $\delta_{\text{GW}}^{2}$ are the between‐ and within‐group variance and h^2^ is the heritability. Given the established very strong genetic basis for the sorts of traits measured here (Gordon et al., 2017; Karino & Haijima, 2001; Lindholm & Breden, 2002; Tripathi et al., 2009), we made the assumption that *h* ^2^ = 1, meaning that all variance is genetic. Choice of a different value for heritability would not have influenced conclusions, which are based on *relative* differences between various types of population pairs. Following Phillimore et al. (2008), we conducted pairwise MANOVA for all sites across watersheds using the R package *stats*. Variance–covariance matrices were then summed to estimate $\delta_{\text{GB}}^{2}$ and $\delta_{\text{GW}}^{2}$.

### 2.4 Comparison of genotypic and phenotypic data

To enable direct comparisons of population structure between the genetic (from both datasets) and phenotypic (male color) data, we analyzed both types of data using discriminant analysis of principal components (DAPC; Jombart, Devillard, & Balloux, 2010) implemented in the R package *adegenet*. This method infers individual exchangeability between sites and allows evolutionary inferences from the classification of each individual into different categories of sites (Hendry, Kaeuffer, Crispo, Peichel, & Bolnick, 2013). For each data type (genetic or phenotypic), we recorded the probability that each individual is assigned to (a) its site of origin, (b) a site from the same watershed at the same elevation (upstream vs. downstream), (c) a site from the same watershed but with a different elevation, (d) a site from the other watershed with the same elevation, and (e) a site from the other watershed with a different elevation. We then recorded “classification” as the highest assignment of an individual to its own site or in any other site, and “cross‐classification” as the highest assignment to any other site apart from the site of origin (Hendry et al., 2013).

We calculated *F*_ST_ (each dataset separately) and *P*_ST_ means and confidence intervals to allow comparisons and used a Mantel test in the R package *vegan* (Oksanen et al., 2018) to statistically compare these measures. Here, we used only *F*_ST_ measures from the 10loci‐20sites dataset, because insufficient overlap occurred between sites in the 42loci‐12sites dataset and the male color dataset.

## 3 RESULTS

We start with a brief summary of the main findings and the analyses supporting them before moving to specific presentation of the specific analyses. Overall, we found strong evidence of gene flow not only within watersheds but also between them—as supported by five analyses. First, structure most strongly supported four clusters for the 10loci‐20sites dataset and three clusters for the 42loci‐12sites dataset, with one of the clusters in each structure analysis including sites from both watersheds. Second, DAPC for the genetic data showed that, although individuals were mostly classified to their site of origin, a reasonable number were classified into sites in the same watershed (especially at the same elevation), and some were even classified into sites in the other watershed (almost exclusively at the same elevation). Third, migrate suggested considerable historical gene flow both within and between watersheds, with estimates between watersheds often higher than some of those within watersheds. Fourth, bayesass suggested very recent migration events among some sites within watersheds, and even from the Paria to Marianne (especially at higher elevations). Fifth, diyabc inferred historical gene flow between the two watersheds at low elevations and recent gene flow between the watersheds at higher elevations. Finally, we found that male color showed little or no correspondence with neutral genetic variation, suggesting that selection tends to erase the effects of gene flow in these particular comparisons.

### 3.1 Genetic variation

In the 10loci‐20sites dataset, 33 out of 128 tests showed departures from HWE after sequential Bonferroni correction (*α* = 0.05). Although these deviations were haphazardly distributed across loci and samples, we nevertheless searched for correlations between *F*_IS_ and *F*_ST_ at the level of individual loci (Waples & Allendorf, 2015). We did not find any positive relationships between these two measures (10loci‐20sites: *r* ^2^ = 0.08; 42loci‐12sites: *r* ^2^ = 0.01), and thus ruled out a Wahlund effect. *F*_IS_ values for *Pre26* were very positive compared with other loci (*Pre26*:* F*_IS_ = 0.23; median for the other loci: *F*_IS_ = 0.04), reflecting heterozygote deficiency. For this locus, micro‐checker indicated potential null alleles in 11 of the 28 samples. This locus was thus excluded from further analyses. In the 42loci‐12sites dataset, 55 out of 798 tests showed departures from HWE after sequential Bonferroni correction. Because these departures only constituted 6% of the tests, and were haphazardly distributed across loci and sites, we did not exclude any loci from this dataset.

In the 10loci‐20sites dataset, 3 out of 1,260 tests showed evidence of linkage disequilibrium after sequential Bonferroni correction. All significant tests were for site P13, which could indicate a small effective population size (*N*_e_), or could show admixture of different lineages for guppies at that site. In the 42loci‐12sites dataset, 15 out of 8,436 tests showed evidence of linkage disequilibrium after sequential Bonferroni correction. However, physical linkage is unlikely given that the loci are known (i.e., specifically developed) to be widely distributed in the guppy genome.

The *F*_ST_ outlier method implemented in lositan detected six loci potentially under selection in the 10loci‐20sites dataset, and 16 loci in the 42loci‐12sites dataset. For the 10loci‐20sites dataset, we did not eliminate any loci, because of low information with only four remaining loci for the analysis. For the 42loci‐12sites dataset, we ran structure with and without the potentially selected loci and obtained the same results. Hence, we kept all loci for subsequent analyses.

The total number of alleles per site ranged from 34 to 141 for the 10loci‐20sites dataset, and from 54 to 262 for the 42loci‐12sites dataset (Supporting Information). Mean number of alleles per locus was 27.11 for the 10loci‐12sites dataset and was 13.02 for the 42loci‐12sites dataset. Observed heterozygosity ranged from 0.292 to 0.752 for the former and from 0.073 to 0.573 for the latter (Supporting Information). Average observed heterozygosity was higher in downstream sites (*H*_o_ = 0.73 ± 0.06; 10loci‐20sites dataset) than in upstream sites (*H*_o_ = 0.54 ± 0.14; 10loci‐20sites dataset). *F*_ST_ values were higher between sites that were geographically more distant (Supporting Information).

### 3.2 Population structure and gene flow

#### 3.2.1 STRUCTURE

**Figure 2:**
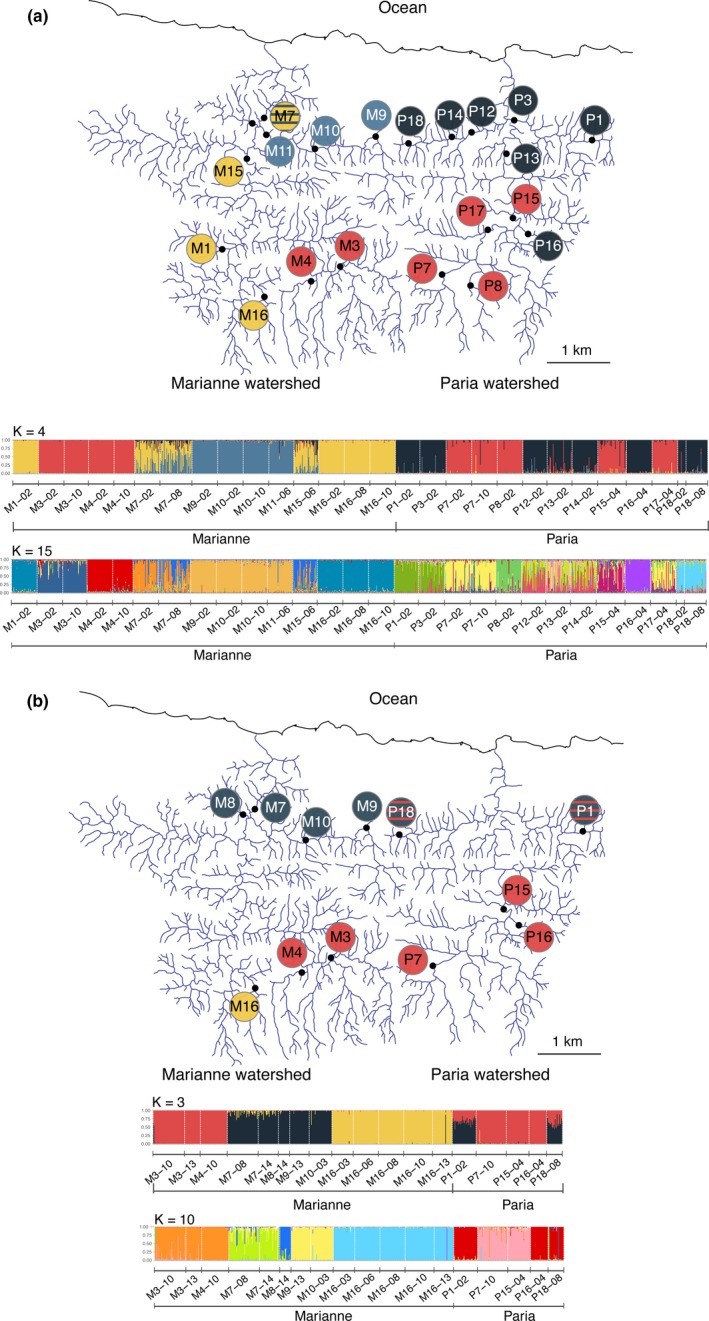
Distribution of clusters inferred by structure analysis and their corresponding maps based on (a) 10loci‐20sites and (b) 42loci‐12sites datasets. For (a), *K* = 4 (mean Δ*K* = 378.42 among three replicates); for (b), *K* = 3 (mean Δ*K* = 87.71 among three replicates). Sites on the map are colored according to the highest assignment to a cluster. When individuals of a site show admixture, site symbol is filled with stripes of the corresponding color. Additional support for *K* = 15 (10loci‐20sites) and *K* = 10 (42loci‐12sites) is also represented

The most likely number of clusters was four for the 10loci‐20sites dataset and three for the 42loci‐12sites dataset (Figure 2 and Supporting Information). Of particular note, both datasets revealed a cluster composed of eastern upstream Marianne sites (M3 and M4) and western upstream Paria sites (P8, P7, P15, and P17 in both datasets; P16 in the 42loci‐12sites dataset). Both datasets also revealed a cluster composed of several western Marianne (M16, M1, and M15) sites. The remaining cluster in the 42loci‐12sites dataset was composed of eastern downstream Marianne sites (M7, M8, M9, M10) and western downstream Paria sites (P1 and P18). This last cluster was further split into two clusters in the 10loci‐20sites dataset: One cluster composed of sites from the downstream Marianne (M9, M10, M11) and the other of sites from the downstream Paria (P1, P3, P12, P13, P14, P16, and P18). For sites that were sampled multiple times, we found consistent patterns between years. Considerable admixture between the clusters was inferred for P1, P18, M7, and M15, and admixture increased between years for M7 in the 10loci‐20sites dataset. Summarizing these patterns, sites did not cluster together exclusively by watershed but rather also according to their geographic position (upstream vs. downstream; and eastern vs. western in the Marianne). We also found moderate support for a structure of 15 clusters for the 10loci‐20sites dataset and 10 clusters for the 42loci‐20sites dataset (Figure 2). These clusters are much more conservative; that is, for both datasets each site often constitutes its own exclusive cluster, with the notable exception of sites located in a portion of the river that is called the “Petite Marianne” (M9, M10, and M11), which cluster together in both datasets.

#### 3.2.2 DAPC

**Figure 3:**
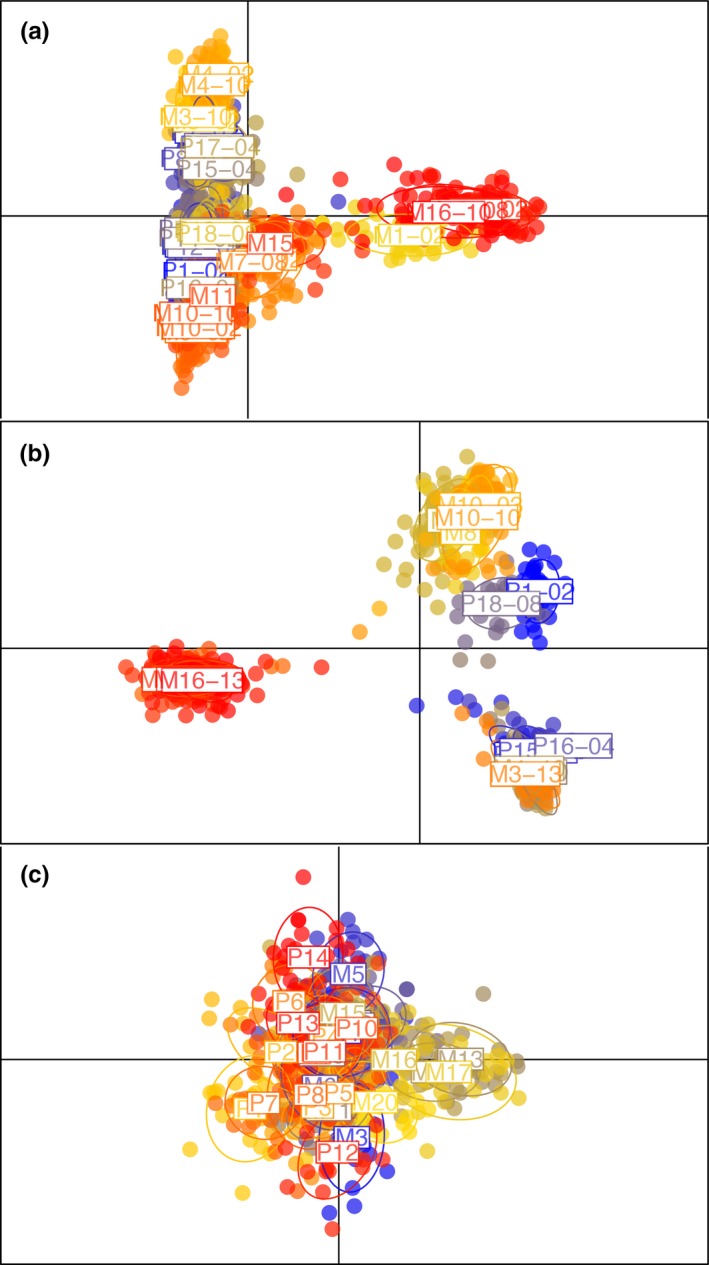
Scatter plot based on discriminant analysis of principal components for (a) 10loci‐20sites neutral markers, (b) 42loci‐12sites neutral markers, and (c) male color traits. Colors correspond to a posteriori groups defined by the DAPC analysis. Individuals are represented as dots and groups as inertia ellipses

**Figure 4:**
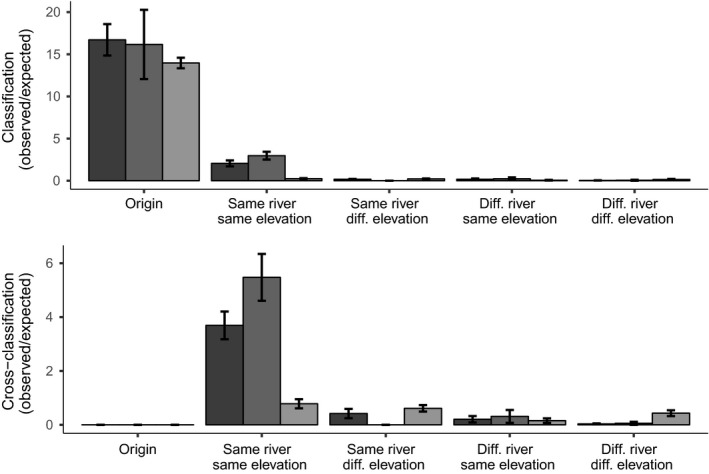
Ratio of the mean number of individuals classified into each category as indicated on the x‐axis to the mean number expected to be classified into these categories by chance. Upper panel shows the classification for the three datasets (10loci‐20sites in dark gray, 42loci‐12sites in medium gray, and male color in light gray), and lower panel shows cross‐classification

For both genetic datasets, classification was highest to the site of origin (Figures 3 and 4), indicating that each site constitutes its own guppy population at least partially isolated from other guppy populations. Some individuals were also assigned to sites from the same watershed at the same elevation, presumably reflecting the easiest contemporary routes for ongoing gene flow. For cross‐classification (i.e., assigning all individuals away from their population of origin), the highest classification was generally to sites in the same watershed at the same elevation, then to the same watershed at a different elevation or instead to the other watershed at the same elevation (Figure 4).

#### 3.2.3 MIGRATE

Both datasets suggested historical gene flow within and between watersheds (Supporting Information). Overall, migration rates were roughly similar among all elevations and between watersheds, suggesting either similar genetic exchange at each of these levels or low power to detect any differences. Despite this absence of large differences in inferred gene flow among site pairs, we draw attention (for the purposes of later discussion) to the relatively high migration rates suggested between the eastern downstream Marianne (M9, M10) and the western downstream Paria (P18, P14, P12, P3, and P1), and between the eastern upstream Marianne (M3, M4) and the western upstream Paria (P7, P8).

#### 3.2.4 BAYESASS

Both datasets suggested reasonable levels of contemporary gene flow between pairs of sites in the same watershed (Supporting Information). Some sites obviously received considerable migrants from neighboring sites; for example (10loci‐20sites) from P1 to P3, P7 to P17, P13 to P12 and P14, P15 to P17, M10 to M9 and M11; and (42loci‐12sites) from P1 to P18, P7 to P15 and P7, P15 to P7, M7 to M8, M8 to M7 and M10, and M10 to M7. Evidence of cross‐watershed contemporary gene flow was also apparent in the 42loci‐12sites dataset, with some sites apparently receiving relatively recent migrants from sites in the adjacent watershed; for example, from P7 to M3 and M4 (upstream Paria to upstream Marianne), from P18 to M7 and M8 (downstream Paria to downstream Marianne), and from M9 to P18 (downstream Marianne to downstream Paria; Supporting Information). We are not certain whether these reflect actual contemporary gene flow events or rather the continued signature of past gene flow events.

#### 3.2.5 DIYABC

**Figure 5:**
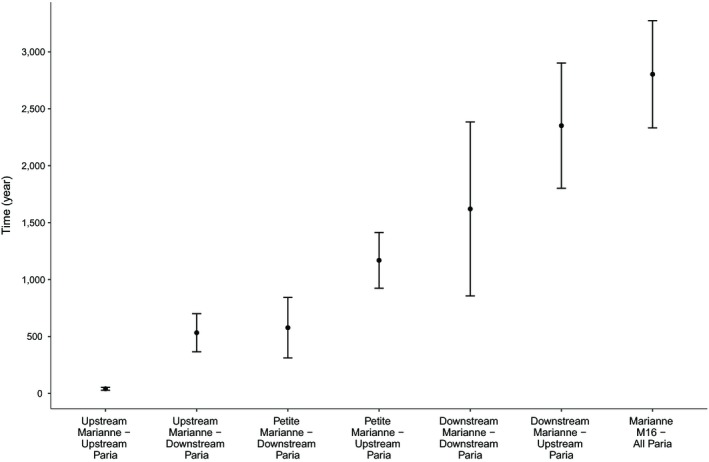
Mean divergence time estimates from pairwise comparison of sites across watersheds, calculated using DIYABC. Errors bars represent standard variation in each group. Groups are as follows: upstream Marianne (M3, M4); upstream Paria (P7, P15); downstream Paria (P1, P16, P18); Petite Marianne (M9, M10); downstream Marianne (M7, M8); Marianne M16 (M16)

Divergence time estimates between watersheds differed greatly among the various pairs of sites (Figure 5). The shortest divergence time (41 ± 13 years) was estimated between nearby sites located in the upstream reaches of the two rivers. The second shortest divergence times were estimated between the downstream Paria and the upstream Marianne (533 ± 167 years) and between the adjacent downstream reaches of the two watersheds (577 ± 265 years). The longest divergence times (2,803 ± 470 years) were estimated between M16 in the western Marianne and various sites in the Paria.

### 3.3 Genetic versus phenotypic patterns

**Figure 6:**
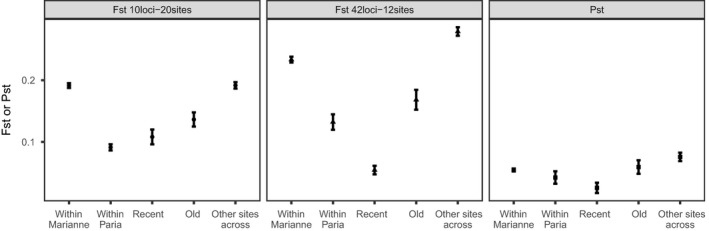
Comparison of the *F*_ST_ values for both genetic datasets and the *P*_ST_ values for the male color traits, within sites located in the Marianne (all sites), within sites located in the Paria (all sites), between sites located in the upstream reached of the watersheds (“recent” gene flow event, between P7 and M3–M4), sites located in the downstream reaches of the watersheds (“old” gene flow event, between P18 and M9–M10), and finally all “other sites across” watersheds

General patterns here were several. First, male color patterns significantly differed between predation regimes (MANOVA; Wilks’ *λ* = 0.766, *df* = 290, *p* < 0.001). Second, classification in DAPC was always highest to the site of origin in all datasets (Figure 4), indicating that each site is a unique “population” to at least some extent. Third, populations differed less phenotypically than genetically at all levels, especially across watersheds (Figures 3 and 6, and Supporting Information). This outcome was mostly driven by variation in neutral genetic differentiation (Figure 6). Together, these results suggest that phenotypic differentiation, while present among all sites, is ultimately more “constrained” in the magnitude of divergence. Third, no correspondence was seen between patterns of neutral genetic differentiation and patterns of phenotypic differentiation (Figure 3), and the comparison between *F*_ST_ and *P*_ST_ matrices was not significant (Mantel test: *r* = 0.17, *p* = 0.27). The only potential indication of an effect of gene flow was that male guppies from sites where we detected a recent gene flow event were very similar in color (Figure 6).

## 4 DISCUSSION

Many studies have emphasized the particular nature of connectivity in streams through ideas such as the “river continuum concept” (Vannote, Minshall, Cummins, Sedell, & Cushing, 1980). Exchanges (of nutrients, individuals, gametes, and genes) along these networks are envisioned to occur primarily within watersheds and to be biased in the downstream direction owing to water flow and barriers such as waterfalls. Genetic data consistent with these interpretations have emerged from a large number of studies of aquatic organisms in rivers, including fishes (e.g., Sivasundar, Bermingham, & Ortí, 2001), aquatic invertebrates (e.g., Monaghan, Spaak, Robinson, & Ward, 2002; Alp, Keller, Westram, & Robinson, 2012), and plants (e.g., Nilsson, Brown, Jansson, & Merritt, 2010). Previous work on guppies has also provided evidence for this type of genetic structure. For example, different watersheds tend to have genetically divergent guppy populations (Alexander et al., 2006; Barson et al., 2009; Carvalho et al., 1991; Suk & Neff, 2009; Willing et al., 2010) and upstream guppy populations show reduced genetic variation consistent with rare colonization events, limitations to upstream gene flow, and possible frequent bottlenecks due to floods (Barson et al., 2009; Crispo et al., 2006; van Oosterhout et al., 2006). At a first cut, our analyses suggest much the same, with the largest among‐site genetic differences occurring between watersheds, with upstream versus downstream populations in a given watershed often differing genetically (Figure 2), and with lower genetic variation in upstream than downstream populations (Supporting Information).

At the same time, our results revealed unexpected levels of cross‐watershed gene flow. Guppy populations in different watersheds were often more closely related genetically than were some guppy populations in the same watershed (Figure 2 and Supporting Information). These findings are consistent with other studies of guppies that have found indications of cross‐watershed gene flow. Some of these linkages can be attributed to known human‐mediated introductions (Shaw, Carvalho, Magurran, & Seghers, 1991), whereas others are more mysterious (Suk & Neff, 2009; Willing et al., 2010). In our case, cross‐watershed gene flow was found in two areas, both closely adjacent tributaries at the same elevation. This finding is reminiscent of a recent study of Atlantic salmon (*Salmo salar*), where fish located at the same elevation but in different rivers were more related to each other than to fish in the same river but at a different elevation (Cauwelier, Stewart, Millar, Gilbey, & Middlemas, 2018). Uniquely in our study, the two cross‐watershed genetic linkages seemed to have occurred on different timescales (historical and contemporary) in different parts of the watersheds—neither of which are associated with any known human‐mediated introductions.

### 4.1 Contemporary and historical cross‐watershed gene flow

We found likely signatures of very recent and probably high gene flow between adjacent headwater tributaries of the Marianne and Paria watersheds. These populations were characterized by very close genetic affinity (Figure 2), high gene flow estimates (Supporting Information), recent estimated dates of divergence (41 ± 13 years; Supporting Information), and even possible ongoing gene flow (from upstream Paria to upstream Marianne; Supporting Information). Of all potential sites for cross‐watershed gene flow, this area is perhaps the least surprising owing to close physical proximity (only a few hundred meters), an only minor elevational barrier, and a well‐travelled road linking them (around 2 km between the two sites). It is particularly tempting to infer human‐mediated causes for the transfer (e.g., we sometimes see children carrying buckets full of guppies), although natural flooding events, perhaps accentuated by deforestation, are a reasonable alternative.

We also found signatures of historical gene flow between the two watersheds. Such signatures have been documented for some other systems and have been attributed to rare and severe events such as ice dams, earthquakes, or volcanic activity (Burridge, Craw, & Waters, 2006, 2007; Gelmond, Hippel, & Christy, 2009; Lescak et al., 2015). In Trinidad's Northern Range, we inferred historical gene flow between one western downstream tributary of the Paria (called the Jordan River) and one eastern downstream tributary of the Marianne (called the Petite Marianne). We suggest, based on several lines of evidence, that the latter was actually colonized at the inferred time from the former. First, Petit Marianne guppies cluster genetically with the adjacent Jordan River guppies. Second, the Petit Marianne is physically isolated by an approximately 10 m waterfall that likely prevents migration from the rest of the Marianne River. Third, guppies are currently found in the headwaters of the Jordan River, less than 50 m of horizontal distance, with an only minor elevational change, from a steep slope down to the Petite Marianne (A. Hendry and P. Bentzen, pers. obs.), which might have allowed Jordan River fish to colonize the Petite Marianne.

Divergence time estimates indicate old (577 years ± 265) connectivity between the Petite Marianne and Jordan River guppies. Several old, but rare, events could explain this historical cross‐watershed linkage. First, indigenous people present on the island since around 1,000 BC could have moved fish from one watershed to the other. However, this explanation seems unlikely given the remote location of these small tributaries, and the fact that indigenous people relied mainly on fish from the ocean rather than fresh water (Newson, 1976). Second, Trinidad is located on the Caribbean tectonic plate, and major earthquakes have been reported since written history of the island (Shepard & Aspinall, 1983). Such earthquakes, violent hurricanes, or massive flooding could have led to river capture (Bishop, 1995), that is, “the transfer of part or all of a (generally well established) river's flow to another river,” causing the movement of Paria guppies from the Jordan River into the Petite Marianne.

Once cross‐watershed gene flow occurs, a logical question is whether that influence spreads far beyond the site of origin. Several studies have shown that experimental introductions of guppies have genetic consequences that spread downstream, including over waterfalls and into different predation environments (Becher & Magurran, 2000; Fitzpatrick et al., 2015; Fraser, Künstner, Reznick, Dreyer, & Weigel, 2015). For our non‐experimental, whether natural or anthropogenic, cross‐watershed transfers, we also see signatures of downstream consequences. For instance, several main‐stem Marianne populations (M7 and M8) immediately below the Petite Marianne show a signature of downstream gene flow from the putative Paria‐origin Petit Marianne fish.

### 4.2 Consequences for adaptive traits

We uncovered signatures of gene flow within and between riverine networks reflecting a complex combination of water flow (biased downstream), barriers (waterfalls), physical proximity, potential recent human introductions, and past geological or climatological events. To what extent has this gene flow influenced adaptive trait variation? A classic theoretical expectation would be reduced divergence in the case of very high, and especially recent, gene flow (Hendry, Day, & Taylor, 2001; Lenormand, 2002). On the other hand, some theoretical treatments suggest a potential positive role for gene flow in facilitating local adaptation (review: Garant et al., 2007).

Previous work on guppies has thus far emphasized strong adaptive divergence among guppy populations in diverse traits such as male color (Endler, 1980; Gotanda & Hendry, 2014; Kemp et al., 2008; Millar et al., 2006), body shape (Burns, Nardo, & Rodd, 2009; Hendry, Kelly, Kinnison, & Reznick, 2006), life history (Reznick, Jv, Rodd, & Ross, 1996), parasite resistance (Fraser & Neff, 2010; Pérez‐Jvostov, Hendry, Fussmann, & Scott, 2015; van Oosterhout, Harris, & Cable, 2003), and behavior (Jacquin et al., 2016; Magurran & Seghers, 1991). Yet a few studies have also hinted that closely adjacent populations can be more phenotypically similar than expected given their environmental differences (Endler, 1978), while others have failed to find such a signature (Fitzpatrick et al., 2015). Given the diverse outcomes of these previous studies, we considered to what extent the patterns of contemporary and historical gene flow we documented might carry over to any signature in adaptive traits, specifically male color.

Overall, male color was quite location‐specific (Figure 4), suggesting adaptation to local conditions. Some of this variation was associated with differences in predation regime (high vs. low) within the Marianne, as described in previous analyses of this system (Gotanda & Hendry, 2014; Millar et al., 2006). Yet considerable variation was also seen between our study sites within a given predation regime (Figures 3 and 4), which previous studies have attributed to these site‐specific factors such as specific predator identities and densities (Millar et al., 2006), canopy cover (Grether et al., 2001; Schwartz & Hendry, 2010), and sexual selection (Schwartz & Hendry, 2007). However, differences among sites in color were generally less than differences among sites in neutral markers (Figure 6 and Supporting Information). This result likely reflects some level of convergent stabilizing selection on male color owing to constraints on the range of possible color space and the need to be attractive to females but also cryptic to predators. By contrast, neutral markers are free to diverge to an extent (mostly) unconstrained by selection, instead being limited only by time and gene flow.

Importantly, we see little evidence that the constraint imposed on divergence for male color reflects gene flow—given the overall lack of correspondence between genetic and phenotypic divergence (Figures 3 and 4; Mantel test: *r* = 0.17, *p* = 0.27). However, one detailed local comparison hinted at potential gene flow effects: Populations from the upstream Paria and upstream Marianne were extremely similar in color (Figure 6). In this particular instance, recent gene flow might have left a signature on male color differentiation. An alternative possibility is that the environments experienced by these two populations were exceptionally similar, and thus favored similar phenotypes, although habitat data do not suggest such exceptional similarity (e.g., Millar et al., 2006). Furthermore, we cannot rule out that gene flow constrains or facilitates adaptation for other traits or in other contexts. For instance, Fitzpatrick et al. (2017) found evidence for trait‐specific constraining and diverging effects in an experimental manipulation of gene flow.

### 4.3 Divergence time

Divergence time between the Paria and Marianne guppies was previously estimated to be approximately 100,000 years based on mitochondrial DNA data (Fajen & Breden, 1992). Our multilocus estimates suggest much more recent connections between the two watersheds, ranging from a maximum of a few thousand years between isolated portions of the watersheds, up to contemporary gene flow between proximate portions of the watersheds at the same elevation. These results have to be tempered because we only tested very simple models of divergence and because homoplasy occurs with microsatellite markers, which could create noise in our results (Estoup, Jarne, & Cornuet, 2002). However, in light of our findings, we would still like to discuss that divergence time between other watersheds extensively studied in Trinidad might also be more recent than previously estimated, a possibility that has important implications for our understanding of adaptive evolution and early speciation in this system. For instance, the general lack of speciation in guppies is often considered surprising (Magurran, 1998) given their ancient divergence—but perhaps gene flow has been much more recent. Also, although we know through experiments that contemporary evolution is common in guppies (Reznick et al., 1990), perhaps even naturally established populations have evolved on much shorter than expected time frames.

## 5 CONCLUSION

Our findings are broadly consistent with previous population genetic work for riverine organisms in general, and for guppies in particular. Specifically, we confirmed within‐watershed gene flow in which upstream populations are less genetically diverse and more isolated than are downstream populations. However, we also discovered levels of cross‐watershed gene flow, to the extent that some populations are more closely related to populations in the adjacent watershed than they are to some populations within their own watershed. Although surprisingly genetic similarities between the Paria and the Marianne watersheds have been previously suggested (Willing et al., 2010), our much more detailed sampling was able to infer where, when, and in what directions these genetic exchanges took place. In one case, cross‐watershed linkages were recent and, in the other case, they occurred centuries ago, suggesting different contributions from geological, climatological, or anthropogenic drivers. However, none of these gene flow patterns seemed to have any major consequence for adaptive trait variation—although our findings do not rule out effects for other traits or on smaller spatial scales. Dispersal, and thus subsequent gene flow, clearly paves the way for colonization of new environments, but it did not seem to here substantially constrain adaptation by guppies to those environments.

## CONFLICT OF INTEREST

None declared.

## AUTHOR CONTRIBUTION

P.B. and A.P.H designed the study; P.B., I.P., and A.P.H contributed to sample collection; L.Ba., J.Q., and I.P genotyped the individuals; J.B.A. made the GIS map; L.Bl. did the analysis and wrote the manuscript.

## Data Availability

Phenotypic data and microsatellite genotypes are accessible from Dryad (https://doi.org/10.5061/dryad.5757d42).

## References

[ece35033-bib-0001] Alexander, H. J. , Taylor, J. S. , Wu, S. S. T. , & Breden, F. (2006). Parallel evolution and vicariance in the guppy (*Poecilia reticulata*) over multiple spatial and temporal scales. Evolution, 60, 2352–2369. 10.1111/j.1365-2427.2012.02758.x 17236426

[ece35033-bib-0002] Alp, M. , Keller, I. , Westram, A. M. , & Robinson, C. T. (2012). How river structure and biological traits influence gene flow: A population genetic study of two stream invertebrates with differing dispersal abilities. Freshwater Biology, 57, 969–981. 10.1111/j.1365-2427.2012.02758.x

[ece35033-bib-0003] Antao, T. , Lopes, A. , Lopes, R. J. , Beja‐Pereira, A. , & Luikart, G. (2008). LOSITAN: A workbench to detect molecular adaptation based on a Fst‐outlier method. BMC Bioinformatics, 9, 323. 18662398 10.1186/1471-2105-9-323 PMC2515854

[ece35033-bib-0004] Avise, J. C. (2000). Phylogeography: The history and formation of species. Cambridge, MA: Harvard University Press.

[ece35033-bib-0005] Barson, N. J. , Cable, J. , & Van Oosterhout, C. (2009). Population genetic analysis of microsatellite variation of guppies (*Poecilia reticulata*) in Trinidad and Tobago: Evidence for a dynamic source‐sink metapopulation structure, founder events and population bottlenecks. Journal of Evolutionary Biology, 22, 485–497. 19210594 10.1111/j.1420-9101.2008.01675.x

[ece35033-bib-0006] Becher, S. A. , & Magurran, A. E. (2000). Gene flow in Trinidadian guppies. Journal of Fish Biology, 56, 241–249.

[ece35033-bib-0007] Beerli, P. (2009). How to use MIGRATE or why are Markov Chain Monte Carlo programs difficult to use? In Bertorelle G., Hauffe H. C., & Bruford M. W. (Eds.), Population genetics for animal conservation (pp. 42–79). Cambridge, UK: Cambridge University Press.

[ece35033-bib-0008] Bishop, P. (1995). Drainage rearrangement by river capture, beheading and diversion. Progress in Physical Geography, 19, 449–473. 10.1177/030913339501900402

[ece35033-bib-0009] Bohonak, A. J. (1999). Dispersal, gene flow, and population structure. The Quarterly Review of Biology, 74, 21–45. 10081813 10.1086/392950

[ece35033-bib-0010] Burns, J. G. , Di Nardo, P. , & Rodd, F. H. (2009). The role of predation in variation in body shape in guppies *Poecilia reticulata*: A comparison of field and common garden phenotypes. Journal of Fish Biology, 75, 1144–1157. 20738605 10.1111/j.1095-8649.2009.02314.x

[ece35033-bib-0011] Burridge, C. P. , Craw, D. , & Waters, J. M. (2006). River capture, range expansion, and cladogenesis: The genetic signature of freshwater vicariance. Evolution, 60, 1038–1049. 16817543

[ece35033-bib-0012] Burridge, C. P. , Craw, D. , & Waters, J. M. (2007). An empirical test of freshwater vicariance via river capture. Molecular Ecology, 16, 1883–1895. 10.1111/j.1365-294X.2006.03196.x 17444899

[ece35033-bib-0013] Carvalho, G. R. , Shaw, P. W. , Magurran, A. E. , & Seghers, B. H. (1991). Marked genetic divergence revealed by allozymes among populations of the guppy *Poecilia reticulata* (Poeciliidae), in Trinidad. Biological Journal of the Linnean Society, 42, 389–405. 10.1111/j.1095-8312.1991.tb00571.x

[ece35033-bib-0014] Castric, V. , Bonney, F. , & Bernatchez, L. (2001). Landscape structure and hierarchical genetic diversity in the brook charr, *Salvelinus fontinalis* . Evolution, 55, 1016–1028. 11430638 10.1554/0014-3820(2001)055[1016:lsahgd]2.0.co;2

[ece35033-bib-0015] Cauwelier, E. , Stewart, D. C. , Millar, C. P. , Gilbey, J. , & Middlemas, S. J. (2018). Across rather than between river genetic structure in Atlantic salmon Salmo salar in north‐east Scotland, UK: Potential causes and management implications. Journal of Fish Biology, 92, 607–620. 10.1111/jfb.13542 29537085

[ece35033-bib-0016] Cornuet, J. M. , Pudlo, P. , Veyssier, J. , Dehne‐Garcia, A. , Gautier, M. , Leblois, R. , … Estoup, A. (2014). DIYABC v2.0: A software to make approximate Bayesian computation inferences about population history using single nucleotide polymorphism, DNA sequence and microsatellite data. Bioinformatics, 30, 1187–1189. 24389659 10.1093/bioinformatics/btt763

[ece35033-bib-0017] Crispo, E. , Bentzen, P. , Reznick, D. N. , Kinnison, M. T. , & Hendry, A. P. (2006). The relative influence of natural selection and geography on gene flow in guppies. Molecular Ecology, 15, 49–62. 10.1111/j.1365-294X.2005.02764.x 16367829

[ece35033-bib-0018] Crookes, S. , & Shaw, P. W. (2016). Isolation by distance and non‐identical patterns of gene flow within two river populations of the freshwater fish *Rutilus rutilus* (L. 1758). Conservation Genetics, 17, 861–874. 10.1007/s10592-016-0828-3 PMC7175732 32355467

[ece35033-bib-0019] Cuenca, A. , Escalante, A. E. , & Pinero, D. (2003). Long‐distance colonization, isolation by distance, and historical demography in a relictual Mexican pinyon pine (*Pinus nelsonii* Shaw) as revealed by paternally inherited genetic markers (cpSSRs). Molecular Ecology, 12, 2087–2097. 12859631 10.1046/j.1365-294x.2003.01890.x

[ece35033-bib-0020] Elphinstone, M. S. , Hinten, G. N. , Anderson, M. J. , & Nock, C. J. (2003). An inexpensive and high‐throughput procedure to extract and purify total genomic DNA for population studies. Molecular Ecology Notes, 3, 317–320. 10.1046/j.1471-8286.2003.00397.x

[ece35033-bib-0021] Endler, J. A. (1977). Geographic variation, speciation, and clines. Monographs in Population Biology, 10, 4504–246. 409931

[ece35033-bib-0022] Endler, J. A. (1978). A predator's view of animal color patterns In Hecht M. K., Steere W. C., & Wallace B. (Eds.), Evolutionary biology (pp. 319–364). Boston, MA: Springer.

[ece35033-bib-0023] Endler, J. A. (1980). Natural selection on color patterns in *Poecilia reticulata* . Evolution, 34, 76. 28563214 10.1111/j.1558-5646.1980.tb04790.x

[ece35033-bib-0024] Estoup, A. , Jarne, P. , & Cornuet, J. M. (2002). Homoplasy and mutation model at microsatellite loci and their consequences for population genetics analysis. Molecular Ecology, 11, 1591–1604. 12207711 10.1046/j.1365-294x.2002.01576.x

[ece35033-bib-0025] Evanno, G. , Regnaut, S. , & Goudet, J. (2005). Detecting the number of clusters of individuals using the software STRUCTURE: A simulation study. Molecular Ecology, 14, 2611–2620. 10.1111/j.1365-294X.2005.02553.x 15969739

[ece35033-bib-0026] Fajen, A. , & Breden, F. (1992). Mitochondrial DNA sequence variation among natural populations of the Trinidad Guppy, *Poecilia reticulata* . Evolution, 46, 1457–1465. 28568990 10.1111/j.1558-5646.1992.tb01136.x

[ece35033-bib-0027] Fitzpatrick, S. W. , Gerberich, J. C. , Kronenberger, J. A. , Angeloni, L. M. , & Funk, W. C. (2015). Locally adapted traits maintained in the face of high gene flow. Ecology Letters, 18, 37–47. 25363522 10.1111/ele.12388

[ece35033-bib-0028] Fitzpatrick, S. W. , Handelsman, C. , Torres‐Dowdall, J. , Ruell, E. , Broder, E. D. , Kronenberger, J. A. , … Funk, W. C. (2017). Gene flow constrains and facilitates genetically based divergence in quantitative traits. Copeia, 105, 462–474.

[ece35033-bib-0029] Francis, R. M. (2017). pophelper: an R package and web app to analyse and visualize population structure. Molecular Ecology Resources, 17, 27–32. 26850166 10.1111/1755-0998.12509

[ece35033-bib-0030] Fraser, B. A. , Künstner, A. , Reznick, D. N. , Dreyer, C. , & Weigel, D. (2015). Population genomics of natural and experimental populations of guppies (*Poecilia reticulata*). Molecular Ecology, 24, 389–408. 10.1111/mec.13022 25444454

[ece35033-bib-0031] Fraser, B. A. , & Neff, B. D. (2010). Parasite mediated homogenizing selection at the MHC in guppies. Genetica, 138, 273–278. 19728113 10.1007/s10709-009-9402-y

[ece35033-bib-0032] Garant, D. , Forde, S. E. , & Hendry, A. P. (2007). The multifarious effects of dispersal and gene flow on contemporary adaptation. Functional Ecology, 21, 434–443.

[ece35033-bib-0033] Gelmond, O. , Von Hippel, F. A. , & Christy, M. S. (2009). Rapid ecological speciation in three‐spined stickleback* Gasterosteus aculeatus* from Middleton Island, Alaska: The roles of selection and geographic isolation. Journal of Fish Biology, 75, 2037–2051. 10.1111/j.1095-8649.2009.02417.x 20738670

[ece35033-bib-0034] Gomez‐Uchida, D. , Knight, T. W. , & Ruzzante, D. E. (2009). Interaction of landscape and life history attributes on genetic diversity, neutral divergence and gene flow in a pristine community of salmonids. Molecular Ecology, 18, 4854–4869. 19878451 10.1111/j.1365-294X.2009.04409.x

[ece35033-bib-0035] Gordon, S. P. , López‐Sepulcre, A. , Rumbo, D. , & Reznick, D. N. (2017). Rapid changes in the sex linkage of male coloration in introduced guppy populations. American Naturalist, 189, 196–200. 10.1086/689864 28107058

[ece35033-bib-0036] Gotanda, K. , Delaire, L. , Raeymaekers, J. , Pérez‐Jvostov, F. , Dargent, F. , Bentzen, P. , … Hendry, A. P. (2013). Adding parasites to the guppy‐predation story: Insights from field surveys. Oecologia, 172, 155–166. 23053240 10.1007/s00442-012-2485-7

[ece35033-bib-0037] Gotanda, K. M. , & Hendry, A. P. (2014). Using adaptive traits to consider potential consequences of temporal variation in selection: Male guppy colour through time and space. Biological Journal of the Linnean Society, 112, 108–122.

[ece35033-bib-0038] Goudet, J. (2005). HIERFSTAT, a package for R to compute and test hierarchical F‐statistics. Molecular Ecology Notes, 5, 184–186.

[ece35033-bib-0039] Grether, G. F. , Hudon, J. , & Millie, D. F. (1999). Carotenoid limitation of sexual coloration along an environmental gradient in guppies. Proceedings of the Royal Society B. Biological Sciences, 266, 1317.

[ece35033-bib-0040] Grether, G. F. , Millie, D. F. , Bryant, M. J. , Reznick, D. N. , & Mayea, W. (2001). Rain forest canopy cover, resource availability, and life history evolution in guppies. Ecology, 82, 1546–1559. 10.1890/0012-9658(2001)082[1546:RFCCRA]2.0.CO;2

[ece35033-bib-0041] Hemmer‐Hansen, J. , Nielsen, E. E. , Frydenberg, J. , & Loeschcke, V. (2007). Adaptive divergence in a high gene flow environment: Hsc70 variation in the European flounder (*Platichthys flesus* L.). Heredity, 99, 592–600. 17848973 10.1038/sj.hdy.6801055

[ece35033-bib-0042] Hendry, A. P. (2016). Eco‐evolutionary dynamics. Princeton, NJ: Princeton University Press.

[ece35033-bib-0043] Hendry, A. P. , Day, T. , & Taylor, E. B. (2001). Population mixing and the adaptive divergence of quantitative traits in discrete populations: A theoretical framework for empirical tests. Evolution, 55, 459–466. 11327154 10.1554/0014-3820(2001)055[0459:pmatad]2.0.co;2

[ece35033-bib-0044] Hendry, A. P. , Kaeuffer, R. , Crispo, E. , Peichel, C. L. , & Bolnick, D. I. (2013). Evolutionary inferences from the analysis of exchangeability. Evolution, 67, 3429–3441. 24299398 10.1111/evo.12160 PMC3852416

[ece35033-bib-0045] Hendry, A. P. , Kelly, M. L. , Kinnison, M. T. , & Reznick, D. N. (2006). Parallel evolution of the sexes? Effects of predation and habitat features on the size and shape of wild guppies. Journal of Evolutionary Biology, 19, 741–754. 10.1111/j.1420-9101.2005.01061.x 16674571

[ece35033-bib-0046] Hoekstra, H. E. , Krenz, J. , & Nachman, M. (2005). Local adaptation in the rock pocket mouse (*Chaetodipus intermedius*): Natural selection and phylogenetic history of populations. Heredity, 94, 217–228. 15523507 10.1038/sj.hdy.6800600

[ece35033-bib-0047] Houde, A. E. , & Endler, J. A. (1990). Correlated evolution of female mating preferences and male color patterns in the guppy *Poecilia reticulata* . Science, 248, 1405–1408. 10.1126/science.248.4961.1405 17747527

[ece35033-bib-0048] Jacquin, L. , Reader, S. M. , Boniface, A. , Mateluna, J. , Patalas, I. , Pérez‐Jvostov, F. , & Hendry, A. P. (2016). Parallel and non‐parallel behavioural evolution in response to parasitism and predation in Trinidadian guppies. Journal of Evolutionary Biology, 29, 1406–1422. 27086945 10.1111/jeb.12880

[ece35033-bib-0049] Jombart, T. , Devillard, S. , & Balloux, F. (2010). Discriminant analysis of principal components: A new method for the analysis of genetically structured populations. BMC Genetics, 11, 4504–15. 10.1186/1471-2156-11-94 PMC2973851 20950446

[ece35033-bib-0050] Karim, N. , Gordon, S. P. , Schwartz, A. K. , & Hendry, A. P. (2007). This is not déjà vu all over again: Male guppy colour in a new experimental introduction. Journal of Evolutionary Biology, 20, 1339–1350. 10.1111/j.1420-9101.2007.01350.x 17584229

[ece35033-bib-0051] Karino, K. , & Haijima, Y. (2001). Heritability of male secondary sexual traits in feral guppies in Japan. Journal of Ethology, 19, 33–37.

[ece35033-bib-0052] Kemp, D. J. , Reznick, D. N. , & Grether, G. F. (2008). Ornamental evolution in Trinidadian guppies (*Poecilia reticulata*): Insights from sensory processing‐based analyses of entire colour patterns. Biological Journal of the Linnean Society, 95, 734–747.

[ece35033-bib-0053] Lenormand, T. (2002). Gene flow and the limits to natural selection. Trends in Ecology & Evolution, 17, 183–189. 10.1016/S0169-5347(02)02497-7

[ece35033-bib-0054] Lescak, E. A. , Bassham, S. L. , Catchen, J. , Gelmond, O. , Sherbick, M. L. , von Hippel, F. A. , & Cresko, W. A. (2015). Evolution of stickleback in 50 years on earthquake‐uplifted islands. Proceedings of the National Academy of Sciences of the United States of America, 112, 201512020. 10.1073/pnas.1512020112 PMC4702987 26668399

[ece35033-bib-0055] Lindholm, A. , & Breden, F. (2002). Sex chromosomes and sexual selection in poeciliid fishes. American Naturalist, 160, S214–S224. 10.1086/342898 18707478

[ece35033-bib-0056] Lippé, C. , Dumont, P. , & Bernatchez, L. (2006). High genetic diversity and no inbreeding in the endangered copper redhorse, Moxostoma hubbsi (Catostomidae, Pisces): The positive sides of a long generation time. Molecular Ecology, 15, 1769–1780. 16689897 10.1111/j.1365-294X.2006.02902.x

[ece35033-bib-0057] Losos, J. B. , Jackman, T. R. , Larson, A. , de Queiroz, K. , & Rodriguez‐Schettino, L. (1998). Contingency and determinism in replicated adaptive radiations of island lizards. Science, 279, 2115–2118. 10.1126/science.279.5359.211 9516114

[ece35033-bib-0058] Magurran, A. E. (1998). Population differentiation without speciation. Philosophical Transactions of the Royal Society B: Biological Sciences, 353, 275–286.

[ece35033-bib-0059] Magurran, A. E. (2005). Evolutionary ecology: The Trinidadian guppy. Oxford, UK: Oxford University Press.

[ece35033-bib-0060] Magurran, A. E. , & Seghers, B. H. (1991). Variation in schooling and aggression amongst guppy (*Poecilia reticulata*) populations in Trinidad. Behaviour, 118, 214–234.

[ece35033-bib-0061] Merilä, J. , & Crnokrak, P. (2001). Comparison of genetic differentiation at marker loci and quantitative traits. Journal of Evolutionary Biology, 14, 892–903. 10.1046/j.1420-9101.2001.00348.x

[ece35033-bib-0062] Millar, N. P. , & Hendry, A. P. (2012). Population divergence of private and non‐private signals in wild guppies. Environmental Biology of Fishes, 94, 513–525.

[ece35033-bib-0063] Millar, N. P. , Reznick, D. N. , Kinnison, M. T. , & Hendry, A. P. (2006). Disentangling the selective factors that act on male colour in wild guppies. Oikos, 113, 4504–12. 10.1111/j.0030-1299.2006.14038.x

[ece35033-bib-0064] Monaghan, M. T. , Spaak, P. , Robinson, C. T. , & Ward, J. V. (2002). Population genetic structure of 3 alpine stream insects: Influences of gene flow, demographics, and habitat fragmentation. Journal of the North American Benthological Society, 21, 114–131. 10.2307/1468304

[ece35033-bib-0065] Newson, L. A. (1976). Aboriginal and Spanish colonial Trinidad : A study in culture contact. Cambridge, MA: Academic Press.

[ece35033-bib-0066] Nilsson, C. , Brown, R. L. , Jansson, R. , & Merritt, D. M. (2010). The role of hydrochory in structuring riparian and Wetland vegetation. Biological Reviews of the Cambridge Philosophical Society, 85, 837–858. 20233190 10.1111/j.1469-185X.2010.00129.x

[ece35033-bib-0067] Oksanen, J. , Blanchet, F. G. , Friendly, M. , Kindt, R. , Legendre, P. , McGlinn, D. , … Wagner, H. (2018). vegan: Community Ecology Package.

[ece35033-bib-0068] Paradis, E. (2010). Pegas: An R package for population genetics with an integrated‐modular approach. 10.1093/bioinformatics/btp696 20080509

[ece35033-bib-0069] Paterson, I. G. , Crispo, E. , Kinnison, M. T. , Hendry, A. P. , & Bentzen, P. (2005). Characterization of tetranucleotide microsatellite markers in guppy (*Poecilia reticulata*). Molecular Ecology Notes, 5, 269–271. 10.1111/j.1471-8286.2005.00895.x

[ece35033-bib-0070] Pérez‐Jvostov, F. , Hendry, A. P. , Fussmann, G. F. , & Scott, M. E. (2015). Testing for local host–parasite adaptation: An experiment with Gyrodactylus ectoparasites and guppy hosts. International Journal for Parasitology, 45, 409–417. 25770861 10.1016/j.ijpara.2015.01.010

[ece35033-bib-0071] Phillimore, A. B. , Owens, I. P. F. , Black, R. A. , Chittock, J. , Burke, T. , & Clegg, S. M. (2008). Complex patterns of genetic and phenotypic divergence in an island bird and the consequences for delimiting conservation units. Molecular Ecology, 17, 2839–2853. 10.1111/j.1365-294X.2008.03794.x 18482263

[ece35033-bib-0072] Pogson, G. H. , Taggart, C. T. , Mesa, K. A. , & Boutilier, R. G. (2001). Isolation by distance in the Atlantic cod, *Gadus morhua*, at large and small geographic scales. Evolution, 55, 131–146. 11263734 10.1111/j.0014-3820.2001.tb01279.x

[ece35033-bib-0073] Primmer, C. R. , Veselov, A. J. , Zubchenko, A. , Poututkin, A. , Bakhmet, I. , & Koskinen, M. T. (2006). Isolation by distance within a river system: Genetic population structuring of Atlantic salmon, *Salmo salar*, in tributaries of the Varzuga River in northwest Russia. Molecular Ecology, 15, 653–666. 10.1111/j.1365-294X.2005.02844.x 16499692

[ece35033-bib-0074] Pritchard, J. K. , Stephens, M. , & Donnelly, P. (2000). Inference of population structure using multilocus genotype data. Genetics, 155, 945–959. 10835412 10.1093/genetics/155.2.945 PMC1461096

[ece35033-bib-0075] R Core Team (2018). R: A language and environment for statistical computing. Vienna, Austria: R Foundation for Statistical Computing.

[ece35033-bib-0076] Rambaut, A. , & Drummond, A. J. (2013). Tracer v1.6.

[ece35033-bib-0077] Räsänen, K. , & Hendry, A. P. (2008). Disentangling interactions between adaptive divergence and gene flow when ecology drives diversification. Ecology Letters, 11, 624–636. 10.1111/j.1461-0248.2008.01176.x 18384363

[ece35033-bib-0078] Raymond, M. , & Rousset, F. (1995). GENEPOP (version 1.2): Population genetics software for exact tests and ecumenicism. Journal of Heredity, 86, 248–249.

[ece35033-bib-0079] Reznick, D. , Bryga, H. , & Endler, J. (1990). Experimentally induced life‐history evolution in a natural population. Nature, 346, 357–359.

[ece35033-bib-0080] Reznick, D. , & Endler, J. (1982). The impact of predation on life history evolution in Trinidadian guppies (*Poecilia reticulata*). Evolution, 36, 160–177. 10.1111/j.1558-5646.1982.tb05021.x 28581096

[ece35033-bib-0081] Reznick, D. N. , Jv, M. J. B. , Rodd, F. H. , & Ross, P. (1996). Life‐history evolution in guppies (*Poecilia reticulata*) 6. Differential mortality as a mechanism for natural selection. Evolution, 50, 1651–1660. 28565709 10.1111/j.1558-5646.1996.tb03937.x

[ece35033-bib-0082] RStudio Team (2016). RStudio: Integrated Development for R. Boston, MA: RStudio, Inc.

[ece35033-bib-0083] Schwartz, A. K. , & Hendry, A. P. (2007). A test for the parallel co‐evolution of male colour and female preference in Trinidadian guppies (*Poecilia reticulata*). Evolutionary Ecology Research, 9, 71–90.

[ece35033-bib-0084] Schwartz, A. K. , & Hendry, A. P. (2010). Testing the influence of local forest canopy clearing on phenotypic variation in Trinidadian guppies. Functional Ecology, 24, 354–364. 10.1111/j.1365-2435.2009.01652.x

[ece35033-bib-0085] Shaw, P. W. , Carvalho, G. R. , Magurran, A. E. , & Seghers, B. H. (1991). Population differentiation in Trinidadian guppies (*Poecilia reticulata*): Patterns and problems. Journal of Fish Biology, 39, 203–209.

[ece35033-bib-0086] Shen, X. , Yang, G. , & Liao, M. (2007). Development of 51 genomic microsatellite DNA markers of guppy (*Poecilia reticulata*) and their application in closely related species: Primer note. Molecular Ecology Notes, 7, 302–306.

[ece35033-bib-0087] Shepard, J. , & Aspinall, W. (1983). Seismicity and earthquake hazard in Trinidad and Tobago, West Indies. Earthquake Engineering and Structural Dynamics, 11, 229–250.

[ece35033-bib-0088] Sivasundar, A. , Bermingham, E. , & Ortí, G. (2001). Population structure and biogeography of migratory freshwater fishes (Prochilodus: Characiformes) in major South American rivers. Molecular Ecology, 10, 407–417. 11298955 10.1046/j.1365-294x.2001.01194.x

[ece35033-bib-0089] Slatkin, M. (1987). Gene flow and the geographic structure of natural populations. Science, 236, 787–792. 3576198 10.1126/science.3576198

[ece35033-bib-0090] Suk, H. Y. , & Neff, B. D. (2009). Microsatellite genetic differentiation among populations of the Trinidadian guppy. Heredity, 102, 425–434. 10.1038/hdy.2009.7 19223925

[ece35033-bib-0091] Taylor, E. B. , & Donald McPhail, J. (2000). Historical contingency and ecological determinism interact to prime speciation in sticklebacks, Gasterosteus. Proceedings of the Royal Society B. Biological Sciences, 267, 2375–2384. 10.1098/rspb.2000.1294 PMC1690834 11133026

[ece35033-bib-0092] Thorpe, R. S. , Malhotra, A. , Black, H. , Daltry, J. C. , & Wuster, W. (1995). Relating geographic pattern to phylogenetic process. Philosophical Transactions of the Royal Society B: Biological Sciences, 349, 61–68. 10.1098/rstb.1995.0091

[ece35033-bib-0093] Travisano, M. , Mongold, J. A. , Bennett, A. F. , & Lenski, R. E. (1995). Experimental tests of the roles of adaptation, chance, and history in evolution. Science, 267, 87–90. 7809610 10.1126/science.7809610

[ece35033-bib-0094] Tripathi, N. , Hoffmann, M. , Willing, E. M. , Lanz, C. , Weigel, D. , & Dreyer, C. (2009). Genetic linkage map of the guppy, Poecilia reticulata, and quantitative trait loci analysis of male size and colour variation. Proceedings of the Royal Society of London. Series B, Biological Sciences, 276, 2195–2208. 19324769 10.1098/rspb.2008.1930 PMC2677598

[ece35033-bib-0095] van Oosterhout, C. , Harris, P. D. , & Cable, J. (2003). Marked variation in parasite resistance between two wild populations of the Trinidadian guppy, *Poecilia reticulata* (Pisces: Poeciliidae). Biological Journal of the Linnean Society, 79, 645–651.

[ece35033-bib-0096] van Oosterhout, C. , Hutchinson, W. F. , Wills, D. P. M. , & Shipley, P. (2004). MICRO‐CHECKER: Software for identifying and correcting genotyping errors in microsatellite data. Molecular Ecology Notes, 4, 535–538. 10.1111/j.1471-8286.2004.00684.x

[ece35033-bib-0097] van Oosterhout, C. , Joyce, D. A. , Cummings, S. M. , Blais, J. , Barson, N. J. , Ramnarine, I. W. , … Cable, J. (2006). Balancing selection, random genetic drift, and genetic variation at the major histocompatibility complex in two wild populations of guppies (*Poecilia reticulata*). Evolution, 60, 2562–2574. 17263117

[ece35033-bib-0098] Vannote, R. L. , Minshall, G. W. , Cummins, K. W. , Sedell, J. R. , & Cushing, C. E. (1980). The river continuum concept. Canadian Journal of Fisheries and Aquatic Sciences, 37, 130–137. 10.1139/f80-017

[ece35033-bib-0099] Waples, R. S. , & Allendorf, F. (2015). Testing for Hardy‐Weinberg proportions: Have we lost the plot? Journal of Heredity, 106, 4504–19. 10.1093/jhered/esu062 25425676

[ece35033-bib-0100] Watanabe, T. , Yoshida, M. , Nakajima, M. , & Taniguchi, N. (2003). Isolation and characterization of 43 microsatellite DNA markers for guppy (*Poecilia reticulata*). Molecular Ecology Notes, 3, 487–490. 10.1046/j.1471-8286.2003.00490.x

[ece35033-bib-0101] Weese, D. J. , Gordon, S. P. , Hendry, A. P. , & Kinnison, M. T. (2010). Spatiotemporal variation in linear natural selection on body color in wild guppies (*Poecilia reticulata*). Evolution, 64, 1802–1815. 20067520 10.1111/j.1558-5646.2010.00945.x

[ece35033-bib-0102] Willing, E. , Bentzen, P. , Van Oosterhout, C. , Hoffmann, M. , Cable, J. , Weigel, D. , & Dreyer, C. (2010). Genome‐wide single nucleotide polymorphisms reveal population history and adaptive divergence in wild guppies. Molecular Ecology, 19, 968–984. 10.1111/j.1365-294X.2010.04528.x 20149094

[ece35033-bib-0103] Wilson, G. A. , & Rannala, B. (2003). Bayesian inference of recent migration rates using multilocus genotypes. Genetics, 163, 1177–1191. 12663554 10.1093/genetics/163.3.1177 PMC1462502

[ece35033-bib-0104] Zhan, L. , Paterson, I. G. , Fraser, B. A. , Watson, B. , Bradbury, I. R. , Nadukkalam Ravindran, P. , … Bentzen, P. (2017). megasat: Automated inference of microsatellite genotypes from sequence data. Molecular Ecology Resources, 17, 247–256. 10.1111/1755-0998.12561 27333119

