# Supplementary material for: Evidence for contemporary and historical gene flow between guppy populations in different watersheds, with a test for associations with adaptive traits
Source: Ecol Evol. 2019 Mar 29;9(8):4504–17. doi: 10.1002/ece3.5033 (PMC6476793; doi:10.1002/ece3.5033)
Supplement: Supplementary file 1 [file ECE3-9-4504-s001.docx]

**Fig. S1** Delta K (difference in the log probability of data between successive K values) for (A) 10loci-20sites dataset and (B) 42loci-12sites dataset.


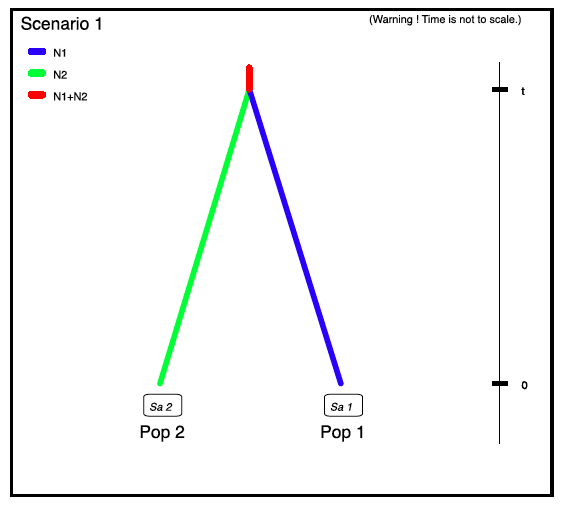


**Fig. S2** Historical model scenario used in DIYABC: two populations of size N1 & N2 have diverged t generations in the past from one population N1+N2. Sa designate samples.

**Fig. S3** Migration rates (M = *m/µ*) among the different parts of the rivers, calculated using Migrate. Sizes of arrow heads represent the variation in the amount of migration; Circles represent sites in the Marianne River, squares represent sites in the Paria river. (A) 10loci-20sites dataset, (B) 42loci-12sites dataset. Arrows between all groups were modelled but are not represented here for clarity of the figure.


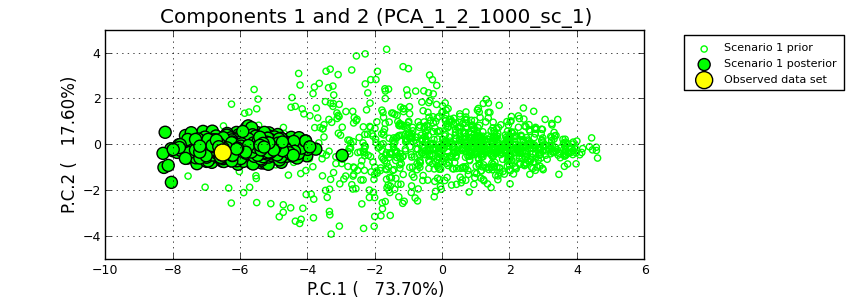


**Fig. S4** PCA plot indicating prior and posterior distributions for the first pairwise divergence estimate – between P& and M3. The yellow dot among the bigger green dots indicate a good fit for the model

**Table S1** List of individuals sampled per site and year in each dataset, as well as allele numbers and observed heterozygosity. All sites are low predation, except when notified (HP for high predation). When a site is present in both datasets, both values of observed heterozygosity and allele numbers are given (first 10loci-20sites, then 42loci-12sites).

| Site – predation regime | 10loci-20sites | 42loci-12sites | Male color | Allele numbers | Observed Heterozygosity |
| --- | --- | --- | --- | --- | --- |
| P1 (2002) | 38 | 38 | 20 | 93 – 134 | 0.632 – 0.354 |
| P2 (2002) | - | - | 20 | - | - |
| P3 (2002) | 41 | - | 20 | 106 | 0.667 |
| P4 (2002) | - | - | 20 | - | - |
| P5 (2002) | - | - | 20 | - | - |
| P6 (2002) | - | - | 18 | - | - |
| P7 (2002)  (2010) | 40  40 | -  48 | 20  - | 87  87 - 180 | 0.702  0.582 – 0.361 |
| P8 (2002) | 40 | - | 20 | 72 | 0.631 |
| P9 (2002) | - | - | 20 | - | - |
| P10 (2002) | - | - | 20 | - | - |
| P11 (2002) | - | - | 20 | - | - |
| P12 (2002) | 38 | - | 20 | 119 | 0.739 |
| P13 (2002) | 39 | - | 20 | 106 | 0.659 |
| P14 (2002) | 40 | - | 20 | 117 | 0.752 |
| P15 (2004) | 45 | 30 | - | 96 - 182 | 0.602 - 0.395 |
| P16 (2004) | 40 | 34 | - | 34 - 54 | 0.292 - 0.073 |
| P17 (2004) | 40 | - | - | 97 | 0.658 |
| P18 (2002)  (2008) | 13  40 | -  25 | -  - | 50  78 - 221 | 0.648  0.685 – 0.480 |
| M1 (2002) | 40 | - | - | 84 | 0.643 |
| M2 (2002) - HP | - | - | 18 | - | - |
| M3 (2002)  (2010)  *(2013)* | 40  38  - | -  50  25 | 20  -  - | 57  52 – 140  101 | 0.568  0.430 – 0.302  0.311 |
| M4 (2002)  (2010) | 40  32 | -  43 | 20  - | 35  38 - 99 | 0.321  0.325 – 0.274 |
| M5 (2002) | - | - | 20 | - | - |
| M6 (2002) | - | - | 20 | - | - |
| M7 (2002) - HP  (2008) - HP  (2014) - HP | 40  51  - | -  49  31 | 20  -  - | 123  141 – 262  206 | 0.732  0.719 – 0.573  0.528 |
| M8 (2014) | - | 18 | - | 158 |  |
| M9 (2002)  (2013) | 40  - | -  31 | 20  - | 89  160 | 0.564  0.386 |
| M10 (2002)  (2003)  (2010) | 40  -  40 | -  36  50 | 20  -  - | 85  183  89 - 184 | 0.616  0.382  0.589 – 0.426 |
| M11 (2006)  (*2002*) | 39  - | -  - | -  19 | -  88 | 0.651  - |
| M13 (2002) - HP | - | - | 20 | - | - |
| M14 (2002) - HP | - | - | 20 | - | - |
| M15 (2002) - HP  (2006) - HP | -  39 | -  - | 20  - | -  121 | -  0.696 |
| M16 (2002)  (2003)  (2006)  (2008)  (2010)  (2013) | 40  -  -  40  40  - | -  34  40  40  46  31 | 20  -  -  -  -  - | 64  130  124  59 - 122  69 – 133  122 | 0.523  0.315  0.327  0.476 – 0.283  0.546 – 0.321  0.338 |
| M17 (2002) - HP | - | - | 20 | - | - |
| M20 (2002) | - | - | 20 | - | - |

**Table S2** Summary of mean and standard deviation for the color traits in each site.

|  | Total number of melanin spots | | Total number of carotenoids spots | | Total number of structural spots | | Total relative area of carotenoids spots | | Total relative area of structural spots | | Total relative area of melanin spots | |
| --- | --- | --- | --- | --- | --- | --- | --- | --- | --- | --- | --- | --- |
| Site | mean | sd | mean | sd | mean | sd | mean | sd | mean | sd | mean | sd |
| M2 | 3.33 | 1.53 | 2.33 | 0.77 | 3.56 | 1.46 | 0.10 | 0.03 | 0.15 | 0.07 | 0.14 | 0.09 |
| M3 | 3.35 | 1.14 | 2.90 | 0.64 | 3.05 | 1.00 | 0.17 | 0.04 | 0.12 | 0.04 | 0.14 | 0.04 |
| M4 | 2.30 | 1.49 | 2.35 | 0.99 | 3.40 | 0.88 | 0.11 | 0.04 | 0.13 | 0.03 | 0.09 | 0.05 |
| M5 | 2.95 | 1.70 | 2.35 | 0.67 | 3.60 | 1.23 | 0.14 | 0.04 | 0.16 | 0.06 | 0.11 | 0.07 |
| M6 | 3.35 | 1.42 | 2.70 | 0.86 | 2.90 | 0.79 | 0.14 | 0.05 | 0.12 | 0.04 | 0.13 | 0.04 |
| M7 | 2.95 | 1.57 | 2.15 | 0.88 | 3.85 | 1.57 | 0.09 | 0.04 | 0.15 | 0.05 | 0.12 | 0.07 |
| M9 | 2.70 | 1.08 | 2.80 | 1.06 | 3.55 | 1.57 | 0.14 | 0.04 | 0.11 | 0.05 | 0.19 | 0.09 |
| M10 | 3.45 | 1.23 | 2.60 | 0.88 | 3.15 | 1.31 | 0.17 | 0.05 | 0.11 | 0.05 | 0.19 | 0.06 |
| M11 | 2.68 | 1.45 | 2.26 | 0.99 | 2.84 | 1.17 | 0.13 | 0.04 | 0.11 | 0.08 | 0.20 | 0.10 |
| M13 | 3.30 | 1.17 | 2.05 | 1.32 | 4.50 | 1.05 | 0.09 | 0.05 | 0.16 | 0.05 | 0.12 | 0.07 |
| M14 | 3.25 | 1.37 | 2.10 | 1.29 | 4.15 | 1.35 | 0.08 | 0.05 | 0.15 | 0.04 | 0.18 | 0.07 |
| M15 | 2.60 | 1.64 | 1.90 | 0.85 | 3.25 | 1.45 | 0.07 | 0.02 | 0.14 | 0.08 | 0.12 | 0.09 |
| M16 | 4.05 | 1.19 | 3.00 | 1.59 | 3.70 | 1.42 | 0.11 | 0.05 | 0.17 | 0.07 | 0.15 | 0.06 |
| M17 | 2.45 | 1.64 | 1.90 | 1.52 | 4.25 | 1.55 | 0.08 | 0.06 | 0.19 | 0.05 | 0.12 | 0.08 |
| M20 | 3.90 | 1.86 | 3.65 | 1.27 | 3.10 | 1.48 | 0.11 | 0.05 | 0.15 | 0.09 | 0.14 | 0.07 |
| P1 | 3.10 | 0.79 | 2.05 | 0.83 | 2.35 | 1.09 | 0.16 | 0.05 | 0.08 | 0.04 | 0.17 | 0.05 |
| P2 | 2.80 | 1.01 | 2.50 | 0.83 | 2.75 | 1.07 | 0.15 | 0.04 | 0.13 | 0.05 | 0.18 | 0.08 |
| P3 | 2.90 | 1.25 | 2.50 | 1.10 | 2.95 | 0.94 | 0.16 | 0.05 | 0.12 | 0.04 | 0.17 | 0.06 |
| P4 | 2.95 | 0.89 | 2.40 | 0.94 | 3.10 | 1.02 | 0.18 | 0.04 | 0.14 | 0.03 | 0.13 | 0.06 |
| P5 | 3.00 | 1.34 | 2.10 | 0.85 | 3.35 | 1.09 | 0.15 | 0.06 | 0.14 | 0.06 | 0.15 | 0.06 |
| P6 | 3.33 | 0.84 | 2.50 | 0.71 | 2.11 | 0.90 | 0.19 | 0.05 | 0.07 | 0.04 | 0.19 | 0.06 |
| P7 | 3.45 | 1.28 | 2.60 | 0.94 | 3.15 | 1.04 | 0.17 | 0.05 | 0.12 | 0.05 | 0.16 | 0.05 |
| P8 | 2.95 | 1.23 | 2.30 | 0.86 | 2.90 | 0.64 | 0.16 | 0.04 | 0.12 | 0.04 | 0.12 | 0.05 |
| P9 | 3.25 | 1.33 | 2.60 | 0.50 | 2.85 | 0.88 | 0.18 | 0.05 | 0.11 | 0.04 | 0.15 | 0.05 |
| P10 | 2.45 | 1.39 | 2.30 | 1.03 | 3.35 | 0.99 | 0.13 | 0.05 | 0.13 | 0.05 | 0.12 | 0.06 |
| P11 | 3.80 | 1.85 | 2.45 | 0.89 | 2.60 | 0.99 | 0.17 | 0.04 | 0.09 | 0.04 | 0.15 | 0.04 |
| P12 | 2.75 | 0.85 | 2.50 | 1.10 | 2.60 | 1.14 | 0.17 | 0.06 | 0.10 | 0.05 | 0.15 | 0.07 |
| P13 | 2.75 | 0.79 | 2.35 | 1.18 | 2.95 | 0.89 | 0.16 | 0.06 | 0.13 | 0.04 | 0.16 | 0.06 |
| P14 | 2.90 | 1.07 | 2.15 | 0.67 | 2.30 | 0.80 | 0.21 | 0.04 | 0.09 | 0.03 | 0.19 | 0.08 |

**Table S3** Heatmap of Pst values for each site in the male color dataset. Values in green represents low phenotypic differentiation, values in red represents higher phenotypic differentiation.

|  | M10 | M11 | M13 | M14 | M15 | M16 | M17 | M2 | M20 | M3 | M4 | M5 | M6 | M7 | M9 |
| --- | --- | --- | --- | --- | --- | --- | --- | --- | --- | --- | --- | --- | --- | --- | --- |
| M11 | 0.064 | - | - | - | - | - | - | - | - | - | - | - | - | - | - |
| M13 | 0.009 | 0.091 | - | - | - | - | - | - | - | - | - | - | - | - | - |
| M14 | 0.001 | 0.056 | 0.002 | - | - | - | - | - | - | - | - | - | - | - | - |
| M15 | 0.066 | 0.001 | 0.093 | 0.061 | - | - | - | - | - | - | - | - | - | - | - |
| M16 | 0.051 | 0.156 | 0.017 | 0.027 | 0.156 | - | - | - | - | - | - | - | - | - | - |
| M17 | 0.011 | 0.012 | 0.029 | 0.014 | 0.015 | 0.076 | - | - | - | - | - | - | - | - | - |
| M2 | 0.000 | 0.052 | 0.009 | 0.002 | 0.055 | 0.049 | 0.008 | - | - | - | - | - | - | - | - |
| M20 | 0.046 | 0.152 | 0.014 | 0.023 | 0.152 | 0.000 | 0.071 | 0.044 | - | - | - | - | - | - | - |
| M3 | 0.000 | 0.073 | 0.007 | 0.001 | 0.074 | 0.050 | 0.013 | 0.000 | 0.044 | - | - | - | - | - | - |
| M4 | 0.065 | 0.001 | 0.094 | 0.055 | 0.003 | 0.164 | 0.009 | 0.052 | 0.160 | 0.077 | - | - | - | - | - |
| M5 | 0.005 | 0.035 | 0.022 | 0.008 | 0.039 | 0.074 | 0.002 | 0.003 | 0.069 | 0.007 | 0.033 | - | - | - | - |
| M6 | 0.004 | 0.037 | 0.021 | 0.007 | 0.041 | 0.071 | 0.003 | 0.002 | 0.066 | 0.006 | 0.035 | 0.000 | - | - | - |
| M7 | 0.003 | 0.025 | 0.016 | 0.006 | 0.029 | 0.057 | 0.002 | 0.002 | 0.052 | 0.004 | 0.022 | 0.000 | 0.000 | - | - |
| M9 | 0.001 | 0.034 | 0.011 | 0.003 | 0.038 | 0.048 | 0.005 | 0.000 | 0.043 | 0.001 | 0.031 | 0.001 | 0.001 | 0.001 | - |
| P1 | 0.140 | 0.004 | 0.159 | 0.102 | 0.001 | 0.233 | 0.031 | 0.113 | 0.232 | 0.162 | 0.013 | 0.088 | 0.090 | 0.054 | 0.069 |
| P10 | 0.044 | 0.002 | 0.072 | 0.042 | 0.004 | 0.135 | 0.005 | 0.036 | 0.131 | 0.052 | 0.000 | 0.021 | 0.023 | 0.015 | 0.022 |
| P11 | 0.004 | 0.025 | 0.019 | 0.008 | 0.029 | 0.064 | 0.001 | 0.003 | 0.058 | 0.006 | 0.022 | 0.000 | 0.000 | 0.000 | 0.001 |
| P12 | 0.065 | 0.000 | 0.093 | 0.057 | 0.001 | 0.160 | 0.011 | 0.053 | 0.157 | 0.075 | 0.000 | 0.035 | 0.037 | 0.024 | 0.034 |
| P13 | 0.051 | 0.002 | 0.079 | 0.046 | 0.005 | 0.147 | 0.005 | 0.041 | 0.143 | 0.060 | 0.001 | 0.024 | 0.026 | 0.016 | 0.024 |
| P14 | 0.120 | 0.005 | 0.144 | 0.095 | 0.002 | 0.214 | 0.031 | 0.099 | 0.213 | 0.135 | 0.013 | 0.077 | 0.079 | 0.052 | 0.065 |
| P2 | 0.065 | 0.003 | 0.094 | 0.053 | 0.006 | 0.167 | 0.006 | 0.050 | 0.164 | 0.078 | 0.001 | 0.030 | 0.032 | 0.018 | 0.028 |
| P3 | 0.023 | 0.009 | 0.048 | 0.025 | 0.012 | 0.106 | 0.001 | 0.018 | 0.101 | 0.028 | 0.006 | 0.008 | 0.009 | 0.006 | 0.010 |
| P4 | 0.027 | 0.017 | 0.055 | 0.027 | 0.021 | 0.122 | 0.000 | 0.020 | 0.118 | 0.034 | 0.014 | 0.007 | 0.009 | 0.005 | 0.010 |
| P5 | 0.023 | 0.014 | 0.049 | 0.024 | 0.018 | 0.111 | 0.000 | 0.017 | 0.106 | 0.028 | 0.011 | 0.007 | 0.008 | 0.004 | 0.009 |
| P6 | 0.073 | 0.001 | 0.099 | 0.057 | 0.003 | 0.170 | 0.009 | 0.057 | 0.168 | 0.086 | 0.000 | 0.036 | 0.039 | 0.022 | 0.032 |
| P7 | 0.000 | 0.068 | 0.010 | 0.002 | 0.070 | 0.056 | 0.011 | 0.000 | 0.051 | 0.000 | 0.072 | 0.005 | 0.004 | 0.003 | 0.001 |
| P8 | 0.048 | 0.004 | 0.076 | 0.043 | 0.006 | 0.144 | 0.004 | 0.038 | 0.140 | 0.057 | 0.001 | 0.021 | 0.023 | 0.014 | 0.022 |
| P9 | 0.015 | 0.033 | 0.040 | 0.017 | 0.037 | 0.105 | 0.001 | 0.010 | 0.100 | 0.020 | 0.032 | 0.002 | 0.002 | 0.001 | 0.004 |

|  | P1 | P10 | P11 | P12 | P13 | P14 | P2 | P3 | P4 | P5 | P6 | P7 | P8 |
| --- | --- | --- | --- | --- | --- | --- | --- | --- | --- | --- | --- | --- | --- |
| M11 | - | - | - | - | - | - | - | - | - | - | - | - | - |
| M13 | - | - | - | - | - | - | - | - | - | - | - | - | - |
| M14 | - | - | - | - | - | - | - | - | - | - | - | - | - |
| M15 | - | - | - | - | - | - | - | - | - | - | - | - | - |
| M16 | - | - | - | - | - | - | - | - | - | - | - | - | - |
| M17 | - | - | - | - | - | - | - | - | - | - | - | - | - |
| M2 | - | - | - | - | - | - | - | - | - | - | - | - | - |
| M20 | - | - | - | - | - | - | - | - | - | - | - | - | - |
| M3 | - | - | - | - | - | - | - | - | - | - | - | - | - |
| M4 | - | - | - | - | - | - | - | - | - | - | - | - | - |
| M5 | - | - | - | - | - | - | - | - | - | - | - | - | - |
| M6 | - | - | - | - | - | - | - | - | - | - | - | - | - |
| M7 | - | - | - | - | - | - | - | - | - | - | - | - | - |
| M9 | - | - | - | - | - | - | - | - | - | - | - | - | - |
| P1 | - | - | - | - | - | - | - | - | - | - | - | - | - |
| P10 | 0.013 | - | - | - | - | - | - | - | - | - | - | - | - |
| P11 | 0.056 | 0.015 | - | - | - | - | - | - | - | - | - | - | - |
| P12 | 0.006 | 0.001 | 0.024 | - | - | - | - | - | - | - | - | - | - |
| P13 | 0.018 | 0.000 | 0.016 | 0.002 | - | - | - | - | - | - | - | - | - |
| P14 | 0.000 | 0.014 | 0.053 | 0.007 | 0.018 | - | - | - | - | - | - | - | - |
| P2 | 0.027 | 0.000 | 0.018 | 0.002 | 0.000 | 0.024 | - | - | - | - | - | - | - |
| P3 | 0.031 | 0.003 | 0.005 | 0.008 | 0.003 | 0.030 | 0.004 | - | - | - | - | - | - |
| P4 | 0.066 | 0.007 | 0.004 | 0.017 | 0.008 | 0.055 | 0.011 | 0.000 | - | - | - | - | - |
| P5 | 0.047 | 0.005 | 0.004 | 0.013 | 0.006 | 0.043 | 0.008 | 0.000 | 0.000 | - | - | - | - |
| P6 | 0.017 | 0.000 | 0.023 | 0.001 | 0.001 | 0.016 | 0.001 | 0.006 | 0.016 | 0.012 | - | - | - |
| P7 | 0.159 | 0.048 | 0.004 | 0.070 | 0.056 | 0.131 | 0.073 | 0.025 | 0.030 | 0.025 | 0.082 | - | - |
| P8 | 0.023 | 0.000 | 0.014 | 0.003 | 0.000 | 0.022 | 0.000 | 0.002 | 0.006 | 0.004 | 0.001 | 0.052 | - |
| P9 | 0.108 | 0.018 | 0.001 | 0.033 | 0.021 | 0.085 | 0.030 | 0.004 | 0.004 | 0.003 | 0.038 | 0.016 | 0.018 |

**Table S4.** Heatmap of paired Fst values for each site in the 10loci-20sites dataset. Values in green represents low genetic differentiation, values in red represents higher genetic differentiation.

|  | P1-02 | | P3-02 | | P7-02 | | P7-10 | | P8-02 | | P12-02 | | P13-02 | | P14-02 | | P15-04 | | P16-04 | | P17-04 | | P18-02 | | P18-08 | | M1-02 | |
| --- | --- | --- | --- | --- | --- | --- | --- | --- | --- | --- | --- | --- | --- | --- | --- | --- | --- | --- | --- | --- | --- | --- | --- | --- | --- | --- | --- | --- |
| P1-02 | - | | - | | - | | - | | - | | - | | - | | - | | - | | - | | - | | - | | - | | - | |
| P3-02 | 0.015 | | - | | - | | - | | - | | - | | - | | - | | - | | - | | - | | - | | - | | - | |
| P7-02 | 0.116 | | 0.084 | | - | | - | | - | | - | | - | | - | | - | | - | | - | | - | | - | | - | |
| P7-10 | 0.118 | | 0.090 | | 0.027 | | - | | - | | - | | - | | - | | - | | - | | - | | - | | - | | - | |
| P8-02 | 0.133 | | 0.100 | | 0.067 | | 0.080 | | - | |  | | - | | - | | - | | - | | - | | - | | - | | - | |
| P12-02 | 0.044 | | 0.028 | | 0.081 | | 0.086 | | 0.093 | | - | | - | | - | | - | | - | | - | | - | | - | | - | |
| P13-02 | 0.038 | | 0.024 | | 0.077 | | 0.086 | | 0.097 | | 0.025 | | - | | - | | - | | - | | - | | - | | - | | - | |
| P14-02 | 0.038 | | 0.024 | | 0.074 | | 0.080 | | 0.087 | | 0.014 | | 0.020 | | - | | - | | - | | - | | - | | - | | - | |
| P15-04 | 0.109 | | 0.083 | | 0.034 | | 0.036 | | 0.044 | | 0.088 | | 0.079 | | 0.077 | | - | | - | | - | | - | | - | | - | |
| P16-04 | 0.236 | | 0.211 | | 0.229 | | 0.202 | | 0.245 | | 0.235 | | 0.238 | | 0.221 | | 0.182 | | - | | - | | - | | - | | - | |
| P17-04 | 0.116 | | 0.086 | | 0.012 | | 0.028 | | 0.052 | | 0.085 | | 0.078 | | 0.075 | | 0.021 | | 0.211 | | - | | - | | - | | - | |
| P18-02 | 0.093 | | 0.074 | | 0.082 | | 0.086 | | 0.088 | | 0.048 | | 0.065 | | 0.052 | | 0.089 | | 0.209 | | 0.092 | | - | | - | | - | |
| P18-08 | 0.088 | | 0.062 | | 0.085 | | 0.086 | | 0.099 | | 0.038 | | 0.052 | | 0.040 | | 0.090 | | 0.222 | | 0.092 | | 0.017 | | - | | - | |
| M1-02 | 0.173 | | 0.153 | | 0.184 | | 0.173 | | 0.201 | | 0.138 | | 0.156 | | 0.144 | | 0.167 | | 0.343 | | 0.168 | | 0.130 | | 0.138 | | - | |
| M3-02 | 0.146 | | 0.113 | | 0.028 | | 0.042 | | 0.087 | | 0.114 | | 0.108 | | 0.103 | | 0.045 | | 0.254 | | 0.027 | | 0.108 | | 0.111 | | 0.218 | |
| M3-10 | 0.187 | | 0.153 | | 0.100 | | 0.075 | | 0.157 | | 0.159 | | 0.160 | | 0.144 | | 0.094 | | 0.307 | | 0.095 | | 0.149 | | 0.159 | | 0.262 | |
| M4-02 | 0.263 | | 0.220 | | 0.153 | | 0.129 | | 0.240 | | 0.234 | | 0.227 | | 0.224 | | 0.143 | | 0.427 | | 0.155 | | 0.218 | | 0.228 | | 0.315 | |
| M4-10 | 0.260 | | 0.219 | | 0.174 | | 0.119 | | 0.248 | | 0.233 | | 0.231 | | 0.218 | | 0.149 | | 0.426 | | 0.167 | | 0.247 | | 0.237 | | 0.310 | |
| M7-02 | 0.090 | | 0.074 | | 0.099 | | 0.105 | | 0.109 | | 0.063 | | 0.076 | | 0.065 | | 0.097 | | 0.255 | | 0.098 | | 0.081 | | 0.084 | | 0.099 | |
| M7-08 | 0.068 | | 0.058 | | 0.078 | | 0.084 | | 0.092 | | 0.052 | | 0.057 | | 0.053 | | 0.075 | | 0.211 | | 0.076 | | 0.069 | | 0.074 | | 0.088 | |
| M9-02 | 0.133 | | 0.125 | | 0.188 | | 0.186 | | 0.204 | | 0.103 | | 0.118 | | 0.119 | | 0.178 | | 0.326 | | 0.183 | | 0.107 | | 0.118 | | 0.219 | |
| M10-02 | 0.109 | | 0.103 | | 0.164 | | 0.162 | | 0.180 | | 0.085 | | 0.098 | | 0.096 | | 0.156 | | 0.307 | | 0.160 | | 0.096 | | 0.103 | | 0.200 | |
| M10-10 | 0.129 | | 0.122 | | 0.179 | | 0.177 | | 0.197 | | 0.097 | | 0.116 | | 0.111 | | 0.170 | | 0.305 | | 0.176 | | 0.099 | | 0.112 | | 0.210 | |
| M11 | 0.098 | | 0.096 | | 0.136 | | 0.139 | | 0.153 | | 0.085 | | 0.086 | | 0.091 | | 0.133 | | 0.276 | | 0.135 | | 0.111 | | 0.112 | | 0.183 | |
| M15 | 0.069 | | 0.055 | | 0.066 | | 0.075 | | 0.079 | | 0.056 | | 0.059 | | 0.053 | | 0.056 | | 0.209 | | 0.062 | | 0.086 | | 0.086 | | 0.061 | |
| M16-02 | 0.242 | | 0.219 | | 0.243 | | 0.233 | | 0.269 | | 0.200 | | 0.223 | | 0.209 | | 0.218 | | 0.409 | | 0.225 | | 0.166 | | 0.185 | | 0.096 | |
| M16-08 | 0.257 | | 0.236 | | 0.261 | | 0.248 | | 0.287 | | 0.219 | | 0.240 | | 0.229 | | 0.235 | | 0.418 | | 0.243 | | 0.193 | | 0.199 | | 0.107 | |
| M16-10 | 0.224 | | 0.202 | | 0.223 | | 0.213 | | 0.249 | | 0.185 | | 0.206 | | 0.196 | | 0.199 | | 0.372 | | 0.209 | | 0.160 | | 0.172 | | 0.081 | |
|  | | M3-02 | | M3-10 | | M4-02 | | M4-10 | | M7-02 | | M7-08 | | M9-02 | | M10-02 | | M10-10 | | M11 | | M15 | | M16-02 | | M16-08 | |  |
| P1-02 | | - | | - | | - | | - | | - | | - | | - | | - | | - | | - | | - | | - | | - | |  |
| P3-02 | | - | | - | |  | | - | | - | | - | | - | | - | | - | | - | | - | | - | | - | |  |
| P7-02 | | - | | - | | - | | - | | - | | - | | - | | - | | - | | - | | - | | - | | - | |  |
| P7-10 | | - | | - | | - | | - | | - | | - | | - | | - | | - | | - | | - | | - | | - | |  |
| P8-02 | | - | | - | | - | | - | | - | | - | | - | | - | | - | | - | | - | | - | | - | |  |
| P12-02 | | - | | - | | - | | - | | - | | - | | - | | - | | - | | - | | - | | - | | - | |  |
| P13-02 | | - | | - | | - | | - | | - | | - | | - | | - | | - | | - | | - | | - | | - | |  |
| P14-02 | | - | | - | | - | | - | | - | | - | | - | | - | | - | | - | | - | | - | | - | |  |
| P15-04 | | - | | - | | - | | - | | - | | - | | - | | - | | - | | - | | - | | - | | - | |  |
| P16-04 | | - | | - | | - | | - | | - | | - | | - | | - | | - | | - | | - | | - | | - | |  |
| P17-04 | | - | | - | | - | | - | | - | | - | | - | | - | | - | | - | | - | | - | | - | |  |
| P18-02 | | - | | - | | - | | - | | - | | - | | - | | - | | - | | - | | - | | - | | - | |  |
| P18-08 | | - | | - | | - | | - | | - | | - | | - | | - | | - | | - | | - | | - | | - | |  |
| M1-02 | | - | | - | | - | | - | | - | | - | | - | | - | | - | | - | | - | | - | | - | |  |
| M3-02 | | - | | - | | - | | - | | - | | - | | - | | - | | - | | - | | - | | - | | - | |  |
| M3-10 | | 0.088 | | - | | - | | - | | - | | - | | - | | - | | - | | - | | - | | - | | - | |  |
| M4-02 | | 0.178 | | 0.232 | | - | | - | | - | | - | | - | | - | | - | | - | | - | | - | | - | |  |
| M4-10 | | 0.195 | | 0.175 | | 0.113 | | - | | - | | - | | - | | - | | - | | - | | - | | - | | - | |  |
| M7-02 | | 0.136 | | 0.181 | | 0.228 | | 0.232 | | - | | - | | - | | - | | - | | - | | - | | - | | - | |  |
| M7-08 | | 0.105 | | 0.142 | | 0.188 | | 0.189 | | 0.021 | | - | | - | | - | | - | | - | | - | | - | | - | |  |
| M9-02 | | 0.217 | | 0.260 | | 0.328 | | 0.323 | | 0.141 | | 0.083 | | - | | - | | - | | - | | - | | - | | - | |  |
| M10-02 | | 0.192 | | 0.236 | | 0.303 | | 0.298 | | 0.117 | | 0.066 | | 0.007 | | - | | - | | - | | - | | - | | - | |  |
| M10-10 | | 0.206 | | 0.248 | | 0.304 | | 0.303 | | 0.128 | | 0.084 | | 0.039 | | 0.036 | | - | | - | | - | | - | | - | |  |
| M11 | | 0.156 | | 0.201 | | 0.257 | | 0.259 | | 0.111 | | 0.066 | | 0.008 | | 0.008 | | 0.042 | | - | | - | | - | | - | |  |
| M15 | | 0.082 | | 0.130 | | 0.172 | | 0.184 | | 0.023 | | 0.017 | | 0.107 | | 0.090 | | 0.103 | | 0.093 | | - | | - | | - | |  |
| M16-02 | | 0.280 | | 0.326 | | 0.393 | | 0.391 | | 0.168 | | 0.138 | | 0.268 | | 0.246 | | 0.256 | | 0.226 | | 0.098 | | - | | - | |  |
| M16-08 | | 0.297 | | 0.341 | | 0.395 | | 0.395 | | 0.186 | | 0.155 | | 0.279 | | 0.260 | | 0.270 | | 0.241 | | 0.111 | | 0.015 | | - | |  |
| M16-10 | | 0.257 | | 0.303 | | 0.348 | | 0.352 | | 0.154 | | 0.127 | | 0.250 | | 0.229 | | 0.239 | | 0.212 | | 0.090 | | 0.019 | | 0.016 | |  |

**Table S5**. Heatmap of paired Fst values for each site in the 42loci-12sites dataset. Values in green represents low genetic differentiation, values in red represents higher genetic differentiation.

|  | P1-02 | P7-10 | P15-04 | P16-04 | P18-08 | M3-10 | M3-13 | M4-10 | M7-08 | M7-14 |
| --- | --- | --- | --- | --- | --- | --- | --- | --- | --- | --- |
| P1-02 | - | - | - | - | - | - | - | - | - | - |
| P7-10 | 0.184 | - | - | - | - | - | - | - | - | - |
| P15-04 | 0.150 | -0.001 | - | - | - | - | - | - | - | - |
| P16-04 | 0.280 | 0.186 | 0.213 | - | - | - | - | - | - | - |
| P18-08 | 0.004 | 0.143 | 0.117 | 0.160 | - | - | - | - | - | - |
| M3-10 | 0.190 | 0.024 | 0.039 | 0.251 | 0.136 | - | - | - | - | - |
| M3-13 | 0.171 | 0.036 | 0.054 | 0.285 | 0.167 | 0.031 | - | - | - | - |
| M4-10 | 0.223 | 0.100 | 0.077 | 0.350 | 0.171 | 0.084 | 0.084 | - | - | - |
| M7-08 | 0.136 | 0.202 | 0.187 | 0.271 | 0.081 | 0.217 | 0.189 | 0.255 | - | - |
| M7-14 | 0.124 | 0.229 | 0.208 | 0.302 | 0.132 | 0.237 | 0.237 | 0.297 | 0.001 | - |
| M8-14 | 0.163 | 0.233 | 0.222 | 0.378 | 0.161 | 0.227 | 0.280 | 0.312 | 0.035 | 0.091 |
| M9-13 | 0.133 | 0.248 | 0.235 | 0.315 | 0.092 | 0.256 | 0.261 | 0.305 | 0.118 | 0.113 |
| M10-03 | 0.113 | 0.260 | 0.244 | 0.293 | 0.152 | 0.263 | 0.262 | 0.330 | 0.107 | 0.133 |
| M10-10 | 0.130 | 0.263 | 0.241 | 0.262 | 0.132 | 0.267 | 0.253 | 0.314 | 0.123 | 0.148 |
| M16-03 | 0.300 | 0.348 | 0.322 | 0.498 | 0.264 | 0.360 | 0.369 | 0.408 | 0.180 | 0.230 |
| M16-06 | 0.289 | 0.340 | 0.307 | 0.460 | 0.244 | 0.356 | 0.347 | 0.392 | 0.176 | 0.217 |
| M16-08 | 0.308 | 0.356 | 0.327 | 0.490 | 0.264 | 0.370 | 0.366 | 0.412 | 0.195 | 0.240 |
| M16-10 | 0.293 | 0.343 | 0.315 | 0.452 | 0.243 | 0.360 | 0.341 | 0.393 | 0.188 | 0.225 |
| M16-13 | 0.283 | 0.336 | 0.310 | 0.488 | 0.264 | 0.343 | 0.366 | 0.392 | 0.178 | 0.228 |

|  | M8-14 | M9-13 | M10-03 | M10-10 | M16-03 | M16-06 | M16-08 | M16-10 | M16-13 |
| --- | --- | --- | --- | --- | --- | --- | --- | --- | --- |
| P1-02 | - | - | - | - | - | - | - | - | - |
| P7-10 | - | - | - | - | - | - | - | - | - |
| P15-04 | - | - | - | - | - | - | - | - | - |
| P16-04 | - | - | - | - | - | - | - | - | - |
| P18-08 | - | - | - | - | - | - | - | - | - |
| M3-10 | - | - | - | - | - | - | - | - | - |
| M3-13 | - | - | - | - | - | - | - | - | - |
| M4-10 | - | - | - | - | - | - | - | - | - |
| M7-08 | - | - | - | - | - | - | - | - | - |
| M7-14 | - | - | - | - | - | - | - | - | - |
| M8-14 | - | - | - | - | - | - | - | - | - |
| M9-13 | 0.155 | - | - | - | - | - | - | - | - |
| M10-03 | 0.168 | -0.019 | - | - | - | - | - | - | - |
| M10-10 | 0.172 | 0.014 | 0.003 | - | - | - | - | - | - |
| M16-03 | 0.278 | 0.308 | 0.332 | 0.324 | - | - | - | - | - |
| M16-06 | 0.252 | 0.302 | 0.319 | 0.319 | 0.009 | - | - | - | - |
| M16-08 | 0.275 | 0.305 | 0.338 | 0.334 | 0.014 | 0.015 | - | - | - |
| M16-10 | 0.242 | 0.302 | 0.323 | 0.323 | 0.010 | 0.010 | 0.013 | - | - |
| M16-13 | 0.283 | 0.296 | 0.320 | 0.311 | 0.022 | 0.024 | 0.012 | 0.015 | - |

**Table S6**. Estimated recent migration rates among sites in the Paria and the Marianne rivers, estimated with BayesAss, from the 10loci-20sites dataset. Values underlined and in bold differ significantly from zero, based on 95% credible intervals. Values in diagonal represent the proportions of non-immigrant individuals at each location.

| To/From | P1 | P3 | P7 | P8 | P12 | P13 | P14 | P15 | P16 | P17 | P18 | M1 | M3 | M4 | M7 | M9 | M10 | M11 | M15 | M16 |
| --- | --- | --- | --- | --- | --- | --- | --- | --- | --- | --- | --- | --- | --- | --- | --- | --- | --- | --- | --- | --- |
| P1 | **0.8854** | 0.0058 | 0.0058 | 0.0057 | 0.0058 | 0.008 | 0.0057 | 0.0058 | 0.0058 | 0.0057 | 0.0058 | 0.0058 | 0.0058 | 0.0058 | 0.0058 | 0.0058 | 0.0082 | 0.0059 | 0.0058 | 0.0058 |
| P3 | **0.1957** | **0.6723** | 0.0056 | 0.0056 | 0.0058 | 0.0283 | 0.0057 | 0.0055 | 0.0056 | 0.0058 | 0.0128 | 0.0056 | 0.0055 | 0.0055 | 0.0056 | 0.0056 | 0.0065 | 0.0056 | 0.0058 | 0.0056 |
| P7 | 0.0055 | 0.0057 | **0.8477** | 0.0067 | 0.0055 | 0.0055 | 0.0055 | 0.0065 | 0.0056 | 0.0054 | 0.0111 | 0.0055 | 0.0309 | 0.0193 | 0.0057 | 0.0056 | 0.0055 | 0.0055 | 0.0056 | 0.0055 |
| P8 | 0.0057 | 0.0056 | 0.0079 | **0.8842** | 0.0055 | 0.0054 | 0.0056 | 0.013 | 0.0055 | 0.0053 | 0.0057 | 0.0055 | 0.0058 | 0.0057 | 0.0058 | 0.0056 | 0.0057 | 0.0056 | 0.0055 | 0.0053 |
| P12 | 0.0108 | 0.0058 | 0.0056 | 0.0056 | **0.6772** | **0.212** | 0.006 | 0.0059 | 0.0056 | 0.0056 | 0.0076 | 0.0057 | 0.0058 | 0.0056 | 0.0057 | 0.0058 | 0.0063 | 0.0057 | 0.0058 | 0.0057 |
| P13 | 0.0157 | 0.0055 | 0.0182 | 0.0059 | 0.0063 | **0.8523** | 0.0057 | 0.021 | 0.0055 | 0.0055 | 0.006 | 0.0057 | 0.0073 | 0.0056 | 0.0056 | 0.0058 | 0.0057 | 0.0055 | 0.0057 | 0.0056 |
| P14 | 0.0063 | 0.0056 | 0.0056 | 0.0055 | 0.0135 | **0.2128** | **0.6734** | 0.0055 | 0.0056 | 0.0055 | 0.0098 | 0.0056 | 0.0056 | 0.0055 | 0.0056 | 0.0055 | 0.0058 | 0.0056 | 0.0063 | 0.0055 |
| P15 | 0.0051 | 0.0052 | 0.0127 | 0.0155 | 0.0051 | 0.0055 | 0.005 | **0.8723** | 0.0103 | 0.0051 | 0.0052 | 0.0051 | 0.0082 | 0.0093 | 0.0051 | 0.0049 | 0.0051 | 0.0051 | 0.0052 | 0.0051 |
| P16 | 0.0054 | 0.0056 | 0.0056 | 0.0054 | 0.0057 | 0.0056 | 0.0054 | 0.0057 | **0.8946** | 0.0055 | 0.0057 | 0.0058 | 0.0055 | 0.0055 | 0.0056 | 0.0053 | 0.0053 | 0.0056 | 0.0055 | 0.0057 |
| P17 | 0.0057 | 0.0055 | **0.1419** | 0.0068 | 0.0055 | 0.0055 | 0.0056 | **0.0874** | 0.0055 | **0.6723** | 0.0054 | 0.0057 | 0.0079 | 0.0057 | 0.0056 | 0.0055 | 0.0056 | 0.0056 | 0.0057 | 0.0054 |
| P18 | 0.0052 | 0.005 | 0.005 | 0.0051 | 0.0052 | 0.0148 | 0.0052 | 0.0048 | 0.005 | 0.005 | **0.8949** | 0.0048 | 0.0049 | 0.0049 | 0.005 | 0.005 | 0.005 | 0.005 | 0.0051 | 0.005 |
| M1 | 0.0055 | 0.0056 | 0.0056 | 0.0054 | 0.0054 | 0.0058 | 0.0057 | 0.0056 | 0.0055 | 0.0056 | 0.0056 | **0.8927** | 0.0055 | 0.0055 | 0.0053 | 0.0058 | 0.0056 | 0.0054 | 0.0057 | 0.0073 |
| M3 | 0.0056 | 0.0055 | 0.009 | 0.0061 | 0.0055 | 0.0055 | 0.0056 | 0.0061 | 0.0054 | 0.0057 | 0.0056 | 0.0056 | **0.889** | 0.0065 | 0.0055 | 0.0057 | 0.0055 | 0.0056 | 0.0055 | 0.0056 |
| M4 | 0.0056 | 0.0055 | 0.0054 | 0.0057 | 0.0056 | 0.0054 | 0.0056 | 0.0055 | 0.0056 | 0.0055 | 0.0055 | 0.0056 | 0.0056 | **0.8943** | 0.0054 | 0.0057 | 0.0057 | 0.0056 | 0.0058 | 0.0054 |
| M7 | 0.0057 | 0.0056 | 0.0057 | 0.0071 | 0.0056 | 0.0071 | 0.0055 | 0.0057 | 0.0055 | 0.0058 | 0.0056 | 0.0075 | 0.0056 | 0.0057 | **0.8647** | 0.0055 | 0.0227 | 0.0057 | 0.012 | 0.0058 |
| M9 | 0.0055 | 0.0054 | 0.0054 | 0.0053 | 0.0054 | 0.0053 | 0.0054 | 0.0055 | 0.0054 | 0.0054 | 0.0054 | 0.0054 | 0.0055 | 0.0054 | 0.0053 | **0.6721** | **0.2304** | 0.0054 | 0.0055 | 0.0055 |
| M10 | 0.0058 | 0.0055 | 0.0056 | 0.0055 | 0.0055 | 0.0056 | 0.0056 | 0.0056 | 0.0057 | 0.0056 | 0.0058 | 0.0056 | 0.0055 | 0.0054 | 0.0056 | 0.0057 | **0.8936** | 0.0055 | 0.0055 | 0.0055 |
| M11 | 0.0057 | 0.0057 | 0.0057 | 0.0056 | 0.0058 | 0.0056 | 0.0057 | 0.0057 | 0.0062 | 0.0056 | 0.0059 | 0.0057 | 0.0057 | 0.0058 | 0.0057 | 0.0056 | **0.2178** | **0.6723** | 0.0125 | 0.0056 |
| M15 | 0.0056 | 0.0059 | 0.0055 | 0.0056 | 0.0057 | 0.0057 | 0.0058 | 0.0056 | 0.0056 | 0.0055 | 0.0067 | 0.006 | 0.0057 | 0.0056 | 0.013 | 0.0056 | 0.0068 | 0.0057 | **0.8829** | 0.0057 |
| M16 | 0.0056 | 0.0056 | 0.0054 | 0.0056 | 0.0056 | 0.0053 | 0.0056 | 0.0056 | 0.0056 | 0.0054 | 0.0054 | 0.006 | 0.0055 | 0.0057 | 0.0055 | 0.0057 | 0.0057 | 0.0057 | 0.0057 | **0.8938** |

**Table S7**. Estimated recent migration rates among sites in the Paria and the Marianne rivers, estimated with BayesAss, from the 42loci-12sites dataset. Values underlined and in bold differ significantly from zero, based on 95% credible intervals. Values in diagonal represent the proportions of non-immigrant individuals at each location.

| To/From | P1 | P7 | P15 | P16 | P18 | M3 | M4 | M7 | M8 | M9 | M10 | M16 |
| --- | --- | --- | --- | --- | --- | --- | --- | --- | --- | --- | --- | --- |
| P1 | **0.9239** | 0.0079 | 0.0066 | 0.0066 | 0.0067 | 0.0067 | 0.0068 | 0.0068 | 0.0067 | 0.0065 | 0.0084 | 0.0065 |
| P7 | 0.0055 | **0.6722** | **0.2649** | 0.0056 | 0.0055 | 0.0131 | 0.0058 | 0.0056 | 0.0055 | 0.0055 | 0.0055 | 0.0054 |
| P15 | 0.0072 | **0.2546** | **0.6738** | 0.0072 | 0.0071 | 0.0072 | 0.0071 | 0.0072 | 0.0072 | 0.0072 | 0.0071 | 0.0071 |
| P16 | 0.0085 | **0.2408** | 0.0083 | **0.6751** | 0.0084 | 0.0083 | 0.0086 | 0.0084 | 0.0084 | 0.0084 | 0.0084 | 0.0084 |
| P18 | **0.2064** | 0.009 | 0.009 | 0.0089 | **0.6756** | 0.009 | 0.0089 | 0.0091 | 0.0103 | **0.0359** | 0.0089 | 0.0089 |
| M3 | 0.0053 | **0.2749** | 0.0053 | 0.0053 | 0.0053 | **0.672** | 0.0053 | 0.0053 | 0.0054 | 0.0053 | 0.0053 | 0.0053 |
| M4 | 0.0061 | **0.2666** | 0.006 | 0.0061 | 0.0062 | 0.006 | **0.6728** | 0.006 | 0.006 | 0.0061 | 0.0061 | 0.006 |
| M7 | 0.0058 | 0.0056 | 0.0054 | 0.0055 | **0.0392** | 0.0054 | 0.0057 | **0.8246** | **0.0299** | 0.0054 | **0.0618** | 0.0057 |
| M8 | 0.0107 | 0.0108 | 0.0108 | 0.0106 | **0.0962** | 0.0108 | 0.0108 | **0.1295** | **0.6774** | 0.0109 | 0.0107 | 0.0108 |
| M9 | 0.0257 | 0.0257 | 0.0259 | 0.0252 | 0.0256 | 0.0257 | 0.0254 | 0.0256 | 0.0257 | **0.6923** | 0.0514 | 0.0258 |
| M10 | 0.0056 | 0.0054 | 0.0051 | 0.0053 | 0.0183 | 0.0055 | 0.0054 | 0.0055 | **0.0367** | 0.0056 | **0.8962** | 0.0053 |
| M16 | 0.0055 | 0.0058 | 0.0057 | 0.0059 | 0.0058 | 0.0058 | 0.0059 | 0.0059 | 0.0173 | 0.0057 | 0.0057 | **0.9252** |

**Table S8**. Mean divergence time estimates from pairwise comparison of locations across watersheds, calculated using DIYABC. 95% credible interval are given in parenthesis.

| Pairwise comparison | Divergence time (years) |
| --- | --- |
| Upstream Marianne - Upstream Paria |  |
| M3-P7  M4-P15  M4-P7  M3-P15 | 26.52 (6.28-74.4) |
|  | 50.4 (8.36-172.8) |
|  | 54 (8.64-194) |
|  | 33.16 (6.76-94.8) |
| Upstream Marianne - Downstream Paria |  |
| M3-P1  M3-P16  M3-P18  M4-P1  M4-P16  M4-P18 | 704 (79.6-3252) |
|  | 303.6 (27.48-1732) |
|  | 532 (59.2-2588) |
|  | 748 (251.2-3644) |
|  | 452 (40-2616) |
|  | 460 (49.2-2404) |
| Petite Marianne - Downstream Paria |  |
| M9-P1  M9-P16  M9-P18  M10-P1  M10-P16  M10-P18 | 265.6 (88.4-988) |
|  | 912 (89.2-4480) |
|  | 652 (94.4-2552) |
|  | 255.6 (32.56-1024) |
|  | 756 (72-3792) |
|  | 624 (86.4-2516) |
| Petite Marianne - Upstream Paria |  |
| M9-P15  M9-P7  M10-P15  M10-P7 | 1316 (170.8-5120) |
|  | 1436 (183.6-5480) |
|  | 988 (120.4-3988) |
|  | 936 (102-4240) |
| Downstream Marianne - Upstream Paria |  |
| M7-P1  M7-P16  M7-P18  M8-P1  M8-P16  M8-P18 | 572 (74.8-2480) |
|  | 1092 (112.4-4880) |
|  | 1296 (724-4200) |
|  | 2296 (1284-6680) |
|  | 2568 (408-7000) |
|  | 1900 (344-5760) |
| Downstream Marianne - Upstream Paria |  |
| M7-P15  M8-P15  M7-P7  M8-P7 | 1812 (266.4-5840) |
|  | 2736 (472-6960) |
|  | 1952 (277.6-6160) |
|  | 2908 (488-7240) |
| Marianne M16 - All Paria |  |
| M16-P1  M16-P15  M16-P16  M16-P18  M16-P7 | 3088 (572-7200) |
|  | 2440 (359.6-6800) |
|  | 2972 (504-7160) |
|  | 3324 (652-7360) |
|  | 2192 (317.2-6640) |
